# Comparative Evaluation of Essential Oils from Medicinal-Aromatic Plants of Greece: Chemical Composition, Antioxidant Capacity and Antimicrobial Activity against Bacterial Fish Pathogens

**DOI:** 10.3390/molecules25010148

**Published:** 2019-12-30

**Authors:** Thekla I. Anastasiou, Manolis Mandalakis, Nikos Krigas, Thomas Vézignol, Diamanto Lazari, Pantelis Katharios, Thanos Dailianis, Efthimia Antonopoulou

**Affiliations:** 1 Institute of Marine Biology, Biotechnology and Aquaculture, Hellenic Centre for Marine Research, 71500 Heraklion, Greece; theanast@hcmr.gr (T.I.A.); t.vezignol_15@envt.fr (T.V.); katharios@hcmr.gr (P.K.); thanosd@hcmr.gr (T.D.); 2 Institute of Plant Breeding and Genetic Resources, Hellenic Agricultural Organization Demeter, P.O. Box 60458, 57001 Thessaloniki, Greece; nikoskrigas@gmail.com; 3 Laboratory of Pharmacognosy, School of Pharmacy, Faculty of Health Sciences, Aristotle University of Thessaloniki, 54124 Thessaloniki, Greece; dlazari@pharm.auth.gr; 4 Laboratory of Animal Physiology, Department of Zoology, School of Biology, Faculty of Sciences, Aristotle University of Thessaloniki, 54124 Thessaloniki, Greece; eantono@bio.auth.gr

**Keywords:** carvacrol, γ-terpinene, Gram-negative, p-cymene, plant extracts

## Abstract

The administration of antibiotics in aquaculture has raised concern about the impact of their overuse in marine ecosystems, seafood safety and consumers’ health. This “green consumerism” has forced researchers to find new alternatives against fish pathogens. The present study focused on 12 Mediterranean medicinal-aromatic plants as potential antimicrobials and antioxidant agents that could be used in fish aquaculture. *In vitro* assays showed that the essential oils (EOs) from all studied plants had anti-bacterial and antioxidant properties, with their efficacy being dependent on their chemical composition. More specifically, EOs rich in carvacrol, p-cymene and γ-terpinene exhibited not only the strongest inhibitory activity against the growth of bacterial pathogens (inhibitory concentration: 26–88 μg mL^−1^), but also the greatest total antioxidant capacity (ABTS: 2591–5879 μmole mL^−1^; CUPRAC: 931–2733 μmole mL^−1^). These compounds were mainly found in the EOs from Greek oregano (*Origanum vulgare* subsp. *hirtum*), Spanish oregano (*Thymbra capitata*) and savoury (*Satureja thymbra*) collected from cultivations in Greece. The specific EOs stand out as promising candidates for the treatment of bacterial diseases and oxidative stress in farmed fish. Further *in vivo* experiments are needed to fully understand the effects of EO dietary supplementation on fish farming processes.

## 1. Introduction

Environment and food safety are of primary importance and there is an increasing concern about the indiscriminate use of antibiotics, both in humans and reared animals. In aquaculture, where high stocking densities, increased temperatures during summer season, nutritional imbalance and handling stress favour the spread of bacterial diseases, the administration of antibiotics is a common practice for mitigating fish morbidity and mortality. Indeed, severe losses in aquaculture worldwide are associated with many opportunistic Gram-negative and Gram-positive bacteria, with pathogens belonging to the genera of *Aeromonas*, *Vibrio*, *Edwardsiella, Flavobacterium*, *Photobacterium*, *Pseudomonas*, *Yersinia, Lactococcus*, *Renibacterium* and *Streptococcus* being among the main aetiological agents [1]. The overuse of antibiotics as a way to treat or prevent diseases in aquaculture settings has led to increased levels of antimicrobial compounds in the surrounding environment, residue accumulation in fish tissues [2,3,4], and modification of the normal microbiota in farmed fish, which can subsequently increase their susceptibility to other infections [5,6]. More importantly, this practice promotes the development of highly antibiotic-resistant bacteria that are increasingly difficult to treat with current antibiotics [7,8].

Since the 1990s, the extensive use of bacterial vaccines in aquaculture has led to the reduction of antibiotics use [9]. However, vaccination has not yet solved the problem of disease outbreaks, as there are only a few registered vaccines and too many pathogens. Moreover, vaccination cannot be applied during the disease-vulnerable early developmental stages of fish, since their immune system is not yet matured [10]. Consequently, there is an urgent need for the adoption of new, eco-friendly, alternative methods for the prevention and treatment of bacterial diseases that can be applicable against a wide range of pathogens in different fish species in aquaculture.

Over the centuries, medicinal-aromatic plants have been readily available, inexpensive and they were widely used as food seasonings (e.g., oregano, rosemary etc.). In addition, they served as a starting material for the production of perfumes, cosmetics and pharmaceuticals [11]. Several medicinal plants and their essential oils (EOs) have been well documented for their antimicrobial properties particularly against human pathogens [12,13]. The EOs are complex mixtures of volatile organic substances with a strong scent, naturally produced by plants as a result of secondary metabolism. Their components are mainly hydrocarbon terpenes/terpenoids and their oxygenated derivatives, such as alcohols, aldehydes, ketones, acids, phenols, ethers and esters, as well as phenylpropanoids and their derivatives [14].

A medicinal-aromatic plant may contain EOs in its flowers, leaves, roots, rhizomes, seeds, fruits, wood and bark [15]. The anti-bacterial, antifungal, antiviral and insecticidal properties of EOs make them an appealing option for the prevention or treatment of infectious diseases in fish farming. Until now, EOs from oregano, rosemary, thyme, laurel, sage, cinnamon, clove, and basil have been used as antimicrobial and antioxidant additives for the preservation of fishery products and the extension of their shelf-life [16]. Furthermore, the addition of essential oils into fish feeds for combating bacterial infections during rearing processes is an emerging research field with great potential [17,18,19]. EOs interact with the cytoplasm and membranes of bacterial pathogens, thus affecting their quorum sensing systems (i.e., bacterial pheromones), inhibiting the production of toxic bacterial metabolites and/or even inhibiting bacterial growth [20,21]. Moreover, EOs are effective natural antioxidants, which are able to compete with synthetic ones [22]. Indeed, they can act as protective agents that prevent or delay the oxidative damage from reactive oxygen species (ROS), which are typically generated by stressful conditions and may seriously affect immune functions of fish [19,23].

The purpose of this study was to comparatively evaluate the *in vitro* anti-bacterial properties of the EOs of 12 medicinal-aromatic plants from the Mediterranean region and determine which of them would be the most effective agents for the mitigation of fish bacterial pathogens in Greek aquaculture. We further evaluated the antioxidant capacity of EOs to determine if these could provide additional benefits to the welfare of reared fishes by helping them to combat oxidative stress. This is one of the most extensive studies dealing with the antioxidant and anti-bacterial capacity of Mediterranean EOs against fish pathogens.

## 2. Results and Discussion

### 2.1. Chemical Composition of Essential Oils

The GC-MS chromatograms of the EOs extracted from the 12 medicinal-aromatic plants are presented in Figure S1 (Supplementary Materials), while the concentrations of individual components and compound classes are summarized in Table 1 and Table S1, respectively. In total, 67 components were identified, which collectively accounted for 83.6–98.0% of the total composition in the analysed EOs. With the exception of lemon balm, which was dominated by sesquiterpene hydrocarbons (53.9%) and lesser amounts of oxygenated monoterpenes (29.6%), all other EOs from Lamiaceae plants were mainly composed of oxygenated monoterpenes (36.0–91.3%) and monoterpene hydrocarbons (1.2–52.2%). The composition of the three Apiaceae plants was highly variable, with fennel and rock samphire consisting almost entirely of oxygenated monoterpenes (95.0%) and monoterpene hydrocarbons (93.9%), respectively, while the wild carrot formed a mixture of monoterpene hydrocarbons (38.3%), sesquiterpene hydrocarbons (30.5%) and oxygenated monoterpenes (14.8%). A distinctly different composition was noticed for chamomile, which represented Asteraceae family in this study. This was the only EO dominated by oxygenated sesquiterpenes (51.3%) followed by sesquiterpene hydrocarbons (40.1%).

**Figure 1:**
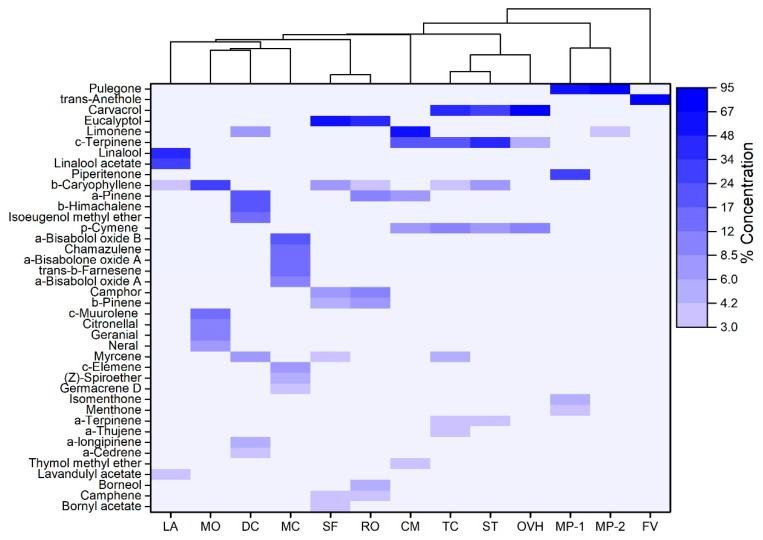
Heatmap and dendrogram of hierarchical cluster analysis for the 13 essential oils under investigation based on the percentage concentrations of their most abundant chemical constituents. (LA = lavender, MO = lemon balm, DC = wild carrot, MC = chamomile, SF = Greek sage, RO = rosemary, CM = rock samphire, TC = Spanish oregano, ST = savoury, OVH = Greek oregano, MP-1 = pennyroyal from Ikaria, MP-2 = pennyroyal from Thessaloniki, FV = fennel).

More pronounced differences were discerned, when the levels of individual constituents were taken into consideration (Table S1). The compositional differences and similarities among the various EOs are illustrated in the dendrogram generated by HCA analysis of concentration data of individual compounds (Figure 1). Among the Lamiaceae family members, Greek sage and rosemary were grouped together as they shared several compounds, with eucalyptol (53.2% and 45.0%, respectively) and camphor (8.1% and 11.5%, respectively) being the main constituents in both. Spanish oregano, savoury and Greek oregano formed a second cluster, mainly due the high abundance of carvacrol in all of them (42.0%, 32.8% and 72.0%, respectively).

Moreover, γ-Terpinene was the most abundant compound in savoury (34.0%) and the second most abundant compound in Spanish oregano (20.5%), while it was also detected in Greek oregano at lower levels (5.3%). Similarly, p-cymene was also a common ingredient in the three aforementioned EOs at comparable levels (6.5–11.9%). Further similarities were identified for Spanish oregano and savoury, which shared additional minor constituents (e.g., α-thujene, α-pinene, myrcene, α-terpinene, linalool, β-caryophyllene). Intriguingly, the two pennyroyals were clustered together, but with a low degree of similarity. Both EOs were dominated by pulegone (47.6% and 87.2%), but the pennyroyal oil from Ikaria island contained high levels of piperitenone (33%), a compound that was completely absent in the pennyroyal oil from Thessaloniki. Lavender oil was found to be rich in linalool (39.1%) and linalool acetate (31.5%), while lemon balm oil contained β-caryophyllene (27.7%), γ-muurolene (12.6%) and citronellal (10.2%) as main components. 

With regard to Apiaceae family, the EO of fennel was almost entirely composed of trans-anethole (95%), while rock samphire oil presented a high abundance of limonene (53.3%) and γ-terpinene (21.4%). The EO of wild carrot demonstrated a relatively higher chemical diversity, with β-himachalene, α-pinene and isoeugenol methyl ether being detected at similarly high levels (21.6%, 20.5% and 14.8%, respectively). Chamomile (Asteraceae) showed a distinctly different composition compared to all other plants. More specifically, the EO of chamomile consisted of α-bisabolol oxide B (23.3%), α-bisabolone oxide A (16.3%), α-bisabolol oxide A (11.7%), chamazulene (16.3%) and trans-β-farnesene (12.6%), all of them being absent in the EOs of the members of other families.

**Figure 2:**
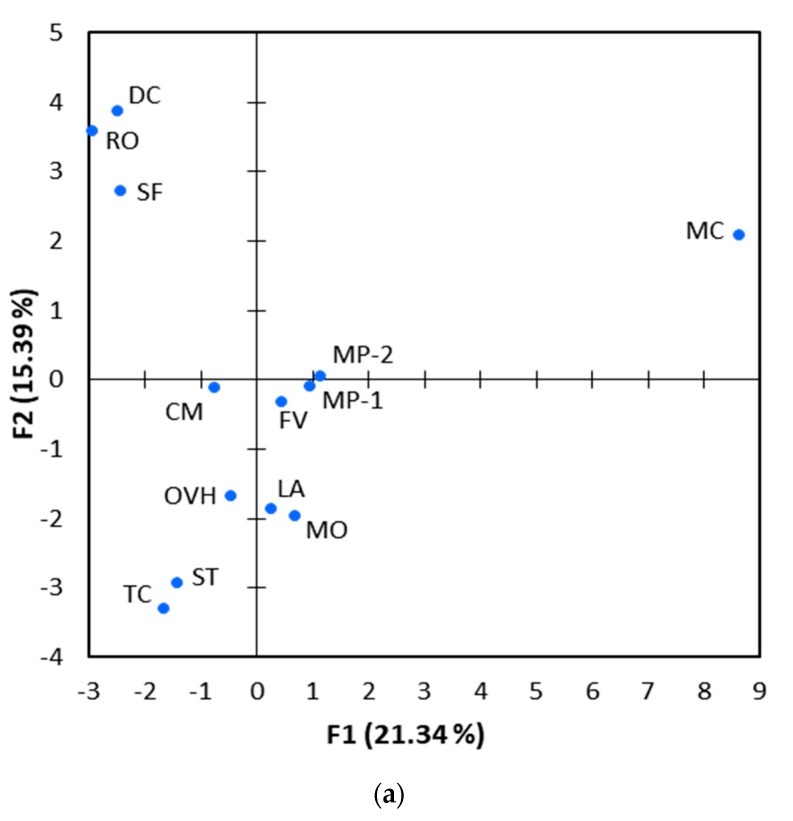

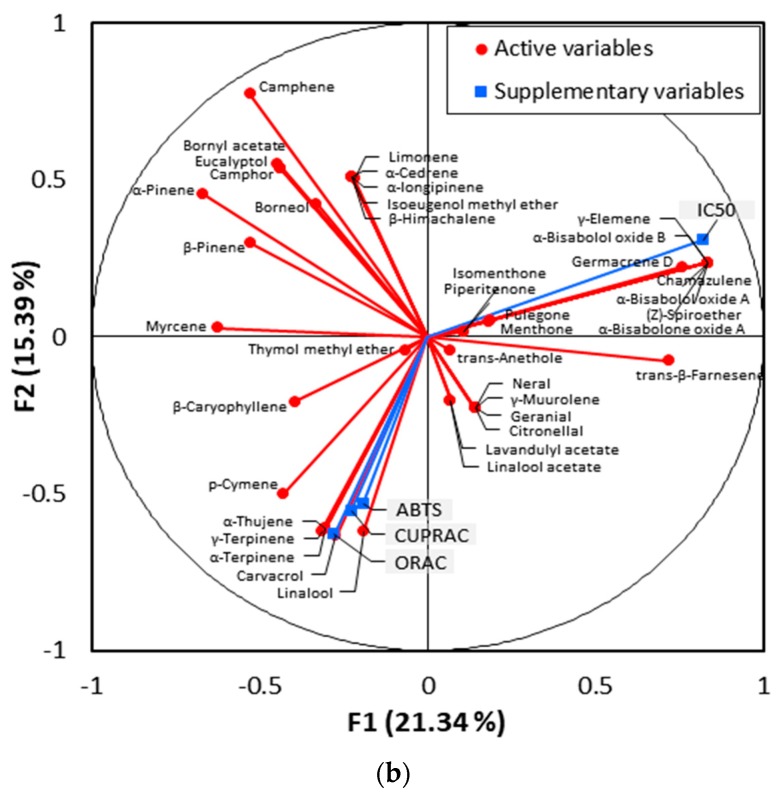
Principal Component Analysis (**a**) scores plot and (**b**) loadings plot obtained from the compositional data of the essential oils under investigation (active variables; red vectors). The anti-bacterial activity (denoted as IC_50_) and the antioxidant capacity determined by CUPRAC, ABTS and ORAC methods are also projected on the loading plot (blue vectors; supplementary variables).

The compositional differences of EOs were further evaluated by PCA analysis. The first two principal components (PC1 and PC2) explained 21.3% and 15.4% of the total variance present in the dataset (Figure 2). They collectively accounted for only 36.7% of the total variance, reflecting the broad dissimilarities in the chemical composition of the 13 EOs under investigation. Despite this fact, the results from PCA were similar to those obtained by HCA analysis (Figure 1). More specifically, Spanish oregano, savoury and Greek oregano were grouped together in the lower left quadrant of the PCA scores plot (Figure 2a), which corresponded to carvacrol, γ-terpinene and p-cymene placement in the loadings plot (Figure 2b). Moreover, Greek sage and rosemary were clustered in the upper left side of the panel, representing EOs rich in eucalyptol, camphor, α-pinene and other compounds. The two pennyroyal oils were also closely located at the centre of the scores plot, which corresponded to pulegone and piperitenone in the loadings plot. It is worth mentioning that chamomile oil was well separated from all other EOs in the scores plot, reflecting the great dissimilarity in its chemical composition. 

The concentration data obtained from the tested EOs were compared with those reported in the literature (Table S2; Supporting Information) and striking similarities were observed for lavender, Greek sage, rosemary and Spanish oregano. Similar to our results, linalool and linalool acetate have been reported to account for over 50% of lavender oil [24,25,26], while carvacrol, γ-terpinene and p-cymene have been shown to represent over 70% of Spanish oregano oil in all previous studies [27,28,29]. Noteworthy similarities were also observed for Greek oregano [30,31,32], and savoury [33,34,35], for which carvacrol, γ-terpinene and p-cymene have been consistently reported among the major constituents. Though, thymol, a frequently detected component of those two EOs (up to 90.2%; [30]), was detected at very low levels in our study (0–2.1%). In line with our results, fennel and pennyroyals have been found to contain trans-anethole (68.5−87.9%; [36,37,38]) and pulegone (19.9−69.2%; [39,40,41]) as the major component, respectively, but considerable differences were noted in the percent contribution of the second most abundant component (e.g., piperitenone of pennyroyals). Less pronounced similarities were noticed for the composition of rock samphire and chamomile, while the greatest dissimilarities were found for lemon balm and wild carrot (Table S2). Though, it should be stressed that compositional differences are reasonable since several factors, such as the part of the plant that is extracted, the growth conditions (e.g., water deficit), the geographical origin, the planting space and time of harvest, the drying methods and extraction processes can affect the final concentrations of compounds in EOs [42,43]. 

### 2.2. Anti-Bacterial Activity of Essential Oils

The anti-bacterial activity of the investigated EOs was evaluated *in vitro* against nine bacterial pathogens isolated from farmed fish and the IC_50_ values are summarized in Table 2. All the bacteria examined in the present study were Gram-negative, which are generally more resistant to antimicrobial compounds than Gram-positive species due to their double-layer cell wall [14,21]. In general, all EOs exhibited antimicrobial activity with the IC_50_ values presenting limited variability among the different fish pathogens. More specifically, the EOs from Greek oregano, savoury and Spanish oregano were considered as very strong anti-bacterials, since they were consistently effective at very low IC_50_ concentrations (within 26–88 μg mL^−1^ range) against all pathogens examined. On the contrary, chamomile oil had moderate antimicrobial activity, as reflected by the higher IC_50_ values (173–985 μg mL^−1^). The rest of EOs exhibited intermediate IC_50_ values and they were regarded as strong or very strong anti-bacterials depending on the bacterial strain investigated.

Concerning bacterial susceptibility, *P. damselae* subsp. *piscicida* (PdKef) was the most sensitive pathogen, as its growth was inhibited at very low concentrations (20–71 μg mL^−1^) by all EOs with the exception of chamomile oil (173 μg mL^−1^). Wild carrot, Greek sage, lemon balm and rosemary oils showed high inhibitory activity against *V. anguillarum* (Vak) (19–71 μg mL^−1^), with the former one being also effective against *E. anguillarum* (EA011113) and *V. harveyi* (VhP1) at low concentrations (32 and 57 μg mL^−1^, respectively). The growth of *E. anguillarum* (EA011113) was also inhibited by lemon balm and rosemary at 53 and 81 μg mL^−1^, respectively. On the contrary, *Vibrio* V1 and *Aeromonas* PDB and NS2 were deemed to be the most resistant bacterial strains, as the inhibition of their growth required, on average, 32 to 72% higher concentration of EOs.

The findings regarding the anti-bacterial activity of EOs are also supported by PCA analysis. Components present in Spanish oregano, savoury and Greek oregano, namely carvacrol, p-cymene, γ-terpinene and α-terpinene, were clustered together on the lower left side of the loadings plot (Figure 2b). These constituents were positively correlated with each other (Spearman’s r ≥ 0.657, *p* < 0.05), but negatively correlated with IC_50_, as shown by its vector in the upper right quadrant (Spearman’s r ≤ −0.597, *p* < 0.05).

The particularly high anti-bacterial activity of Spanish oregano, savoury and Greek oregano against all examined bacterial strains could be attributed to carvacrol, which was the major component shared between the specific EOs and a compound that has been reported to exert potent outer membrane disintegrating properties [44]. In particular, the exposure to carvacrol has been shown to induce striking structural changes on the surface of Gram-negative cells that were deemed responsible for bacterial growth inhibition [45]. With regard to the other major constituents of the aforementioned three EOs (i.e., terpenes like p-cymene, limonene, terpinene, sabinene and pinene), limited anti-bacterial activity has been observed *in vitro* when used in their pure form [46,47].

On the contrary, the monoterpene p-cymene (a biological precursor of carvacrol) has been demonstrated in vitro to exert a synergistic effect on the antimicrobial activity of carvacrol [48]. Moreover, the cyclic monoterpene hydrocarbons α- and γ-terpinene are deemed to play an important intermediary role in EOs anti-bacterial activity, as they can be easily oxidized to p-cymene [22], the subsequent hydroxylation of which can lead to the biosynthesis of carvacrol [49,50]. In this context, the prominent anti-bacterial activity observed for Spanish oregano, savoury and Greek oregano may result from the additive or synergistic antimicrobial effects of carvacrol, p-cymene and γ-terpinene, which are all important components in the specific EOs (Figure 1).

The beneficial antimicrobial activity of the Lamiaceae family, and of carvacrol in particular, is widely recognized, but there are also studies suggesting that carvacrol may pose toxicity to fish species [51,52]. However, these studies investigated the toxicity effects arising from mixtures of carvacrol with other compounds or from carvacrol-rich EOs, rather than from carvacrol in pure form. Nevertheless, the potential toxic effects of EOs and/or its chemical components on organisms are dose-dependent [17,18,19,53], while synergistic or antagonistic effects with other components could also play a significant role to the overall toxicity. In this context, it is quite likely that the positive effect of EOs administration on fish (anti-bacterial activity against pathogens) can be evident at much lower concentrations than their respective toxicity thresholds.

### 2.3. Antioxidant Capacity of Essential Oils

The antioxidant activity of the 13 essential oils under investigation was determined by using three different methods: CUPRAC, ABTS and ORAC. In essence, the specific methods measure the ability of compounds to scavenge different types of radicals by HAT (i.e., oxygen radicals by ORAC) and SET mechanisms (i.e., hydroxyl radicals by CUPRAC) or by a combination of them (i.e., cation radicals by ABTS assay). To facilitate a direct comparison of data collected from different EOs and assays, the total antioxidant capacity measured for EOs was compared to that of Trolox standards and the final results were expressed as Trolox equivalent antioxidant capacity (TEAC). The TEAC values obtained from EOs using the different assays are summarized in Table 3.

The results obtained from all three assays demonstrated significant correlations and this was particularly evident for ABTS and CUPRAC (r^2^ > 0.84, *p* < 0.001). The correlations of ORAC data with those derived from ABTS (r^2^ > 0.69) and CUPRAC assay (r^2^ > 0.76) were relatively lower, but they were also of high statistical significance (*p* < 0.001). Among the different EOs, Spanish oregano, savoury and Greek oregano showed the highest TEAC values in all three assays, highlighting their superior potency as antioxidant agents. It is worth mentioning that the TEAC values measured for these three EOs using ABTS (2591–5879 μmol mL^−1^) and CUPRAC assay (931–2733 μmol mL^−1^) were one to three orders of magnitude higher compared to the other EOs (ABTS: 2.7–154 μmol mL^−1^; CUPRAC: 13.1–184 μmol mL^−1^), while the difference was less striking (30–80%) when considering the ORAC data.

The superior antioxidant potency of Spanish oregano, savoury and Greek oregano was also clearly evident from PCA analysis. These three EOs were clustered together in the lower left quadrant of the scores plot (Figure 2a), which coincided with the positioning of ABTS, CUPRAC and ORAC variables in the loadings plot (Figure 2b). The latter were closely located to carvacrol, α-terpinene, γ-terpinene, α-thujene, linalool and p-cymene, the majority of them being the main components of Spanish oregano, savoury and Greek oregano. This overlap was indicative of a close relationship between the specific compounds and antioxidant capacity of EOs. Indeed, the concentration of carvacrol demonstrated a strong positive correlation with the antioxidant capacity of EOs (Spearman’s r ≥ 0.732, *p* < 0.05), regardless of the antioxidant assay employed. With regard to the other five components, less strong correlations with antioxidant capacity were revealed (Spearman’s r ≥ 0.507), but the majority of them were still of statistical significance.

Overall, the PCA analysis confirmed that the antioxidant potency of EOs was primarily associated with the presence of specific chemical compounds rather than the entire range of constituents. According to Ruberto and Baratta (2000), carvacrol is one of the most active oxygenated monoterpenes and it is responsible for the antioxidant activity of many EOs [54]. The same applies for α-terpinene and γ-terpinene, which stand out as the most active members among monoterpene hydrocarbons and exhibit similar antioxidant effectiveness comparable to carvacrol. Moreover, De Oliveira et al. (2015) have demonstrated that p-cymene is a monoterpene with high antioxidant potential, as it inhibits the formation of lipid and nitrite radicals and helps to reduce lipid peroxidation and nitrite content in the hippocampus of adult mice [55]. These findings are also supported by Amorati et al. (2013) who postulated that EOs enriched in phenolics and cyclohexadiene-like compounds (e.g., γ-terpinene) should be effective antioxidants [56]. In this context, it is reasonable to conclude that the high antioxidant capacity observed in the present study for Spanish oregano, savoury and Greek oregano actually resulted from the prominent presence of carvacrol, p-cymene, α-terpinene and γ-terpinene in these EOs.

## 3. Materials and Methods

### 3.1. Plant Material and Extraction of Essential Oils

All EOs employed in the current study were provided by Dioscurides Co. (Anarrachi, Ptolemaida-Kozani, Greece), Icaronix (Ikaria Island, Greece) and Vessel Essential Oils (Neo Rysio, Thessaloniki, Greece) companies. They were industrially extracted from air-dried aerial parts of 12 medicinal-aromatic plants, cultivated in northern Greece (Thessaloniki, Ptolemaida, Grevena) and Ikaria Island in south-eastern Greece, with the exception of wild carrot which was wild-harvested (Table 4). These included eight perennials from the Lamiaceae family (*Lavandula angustifolia* ‘Hemus’, *Melissa officinalis*, *Mentha pulegium* from both Thessaloniki and Ikaria island, *Origanum vulgare* subsp. *hirtum*, *Rosmarinus officinalis*, *Satureja thymbra*, *Thymbra capitata* and *Salvia fruticosa*), three perennials from the Apiaceae family (*Crithmum maritimum, Daucus carota*, seeds of *Foeniculum vulgare*) and one annual herb from the Asteraceae family (*Matricaria chamomila*). 

### 3.2. Gas Chromatography-Mass Spectrometry Analysis

The EOs extracted from the 12 medicinal-aromatic plants were analyzed by GC-MS to determine their chemical composition. The EOs were diluted with hexane (1:10, *v*/*v*) and 1-μL aliquots were injected onto a GCMS-QP2010 (Shimadzu, Kyoto, Japan) gas chromatography-mass spectrometry system to determine their chemical composition. Separation of the compounds was performed on a HP-5 MS capillary column (30 m × 0.25 mm i.d., film thickness 0.25 μm; Agilent Technologies, Santa Clara, CA, USA) using helium as a carrier gas at a flow rate of 1.0 mL min^−1^. The column temperature was programmed to ramp from 50 °C to 290 °C at a rate of 4 °C min^−1^. The injector was set at 230 °C and operated in split mode (split ratio = 1:10), while the GC–MS transfer line and the ion source were set at 300 °C and 230 °C, respectively. The mass spectrometer was operated in the electron ionization mode (70 eV) and full-scan mass spectra were acquired from *m*/*z* 100 to 600. 

Arithmetic indices for all compounds were determined according to van Den Dool and Kratz (1963) [57], using n-alkanes as standards. The identification of the components was based on comparison of their mass spectra with those present in NIST21 and NIST107 mass spectral databases [58], and by comparison of their retention indices with literature data [59]. The identity of several components in EOs was further confirmed by co-chromatography with authentic compounds.

### 3.3. Evaluation of Anti-Bacterial Activity

Anti-bacterial activity of EOs was evaluated against nine fish bacterial pathogens, which have been isolated during fish disease outbreaks in various aquaculture sites around Greece over the last decade (Table 5). Six of those species have been fully sequenced while the rest have been identified using biochemical, serological and molecular tools. The four strains of *Aeromonas veronii bv. sobria* (NS2, NS22, PDB and NS13) were isolated from European seabass (*Dicentrarchus labrax*) [60], *Edwardsiella anguillarum* (EA011113) was isolated from sharpsnout seabream (*Diplodus puntazzo*) [61,62], *Vibrio alginolyticus* (V1) from gilthead seabream (*Sparus aurata*) [63], *Vibrio harveyi* (VhP1) and *Vibrio anguillarum* (Vak) from European seabass and *Photobacterium damselae* subsp. *piscicida* (PDKef) from common seabream (*Pagrus pagrus*). 

The ability of EOs to inhibit the growth of bacterial pathogens was evaluated using the broth microdilution method, as previously described [64]. In brief, a bacterial culture was prepared for each pathogen using the respective culture medium at 2× concentration and turbidity was adjusted to 0.5 McFarland (i.e., ∼10^8^ CFU mL^−1^). The bacterial suspension was further diluted 1:100 with 2× culture medium to achieve a cell density of ∼10^6^ CFU mL^−1^. The EOs were dissolved in dimethylsulphoxide (DMSO) to prepare stock solutions of 2% (*v*/*v*) and further diluted 1:10 (*v*/*v*) with water to prepare the working solutions (final concentration of approximately 1800 μg mL^−1^). To ensure homogeneity, the aqueous solutions were placed in a sonication bath for 30 s. Some EOs formed homogeneous emulsions that remained stable during extended refrigerated storage. Subsequently, 50-μL aliquots of each working solution were loaded on 384-well polystyrene microplates and serial two-fold dilutions in water were carried out in triplicate, followed by the addition of diluted bacterial suspension (50 μL). A total of 11 serial dilutions were performed and the final concentrations of essential oils in microplate wells ranged from 0.4 to 900 μg mL^−1^.

Mixing of the assay components (i.e., bacterial suspension, essential oil solutions, water) in microplates and preparation of serial dilutions were performed by an automated liquid handling system (Biomek 2000; Beckman Coulter, Fullerton, CA, USA). Microplates were incubated at 25 °C for 22 h and bacterial growth in each microculture was monitored by measuring optical density at 600 nm (OD600) every 20 min using a microplate reader (Infinite F200 PRO, Tecan GmbH, Grödig, Austria). The area under the growth curve (i.e., OD600 vs. time) was integrated for each microdilution assay and the data were used for estimating half maximal concentration of each essential oil inhibiting 50% of bacterial growth (IC_50_). All experiments were performed in triplicate and the average IC_50_ of each EO was derived for each bacterial isolate. Growth controls (cell culture without EOs) and sterility controls (growth medium without cells) were also included in every microplate that was assayed. In addition, treatment of bacterial cells with florfenicol and oxytetracycline, two standard antibiotics that are commonly administered to farmed fishes, were used as positive controls. A total of 4320 microcultures were performed in this study.

### 3.4. Evaluation of Antioxidant Capacity

The antioxidant capacity of EOs was firstly evaluated using the oxygen radical absorbance capacity (ORAC) assay, which is based on hydrogen atom transfer (HAT-based) reaction mechanism. The assay included the preparation of a 75 mM phosphate buffer (pH 7.4), a 7% *w*/*v* solution of randomly methylated β-cyclodextrin (RMCD) in ethanol, as well as the preparation of a 300 mM 2,2’-azobis(2-amidinopropane) dihydrochloride (AAPH) solution and a 0.05 μM fluorescein solution (both in 75 mM phosphate buffer). The EOs were initially dissolved in ethanol to make 2% (*v*/*v*) solutions and then subjected to 2-fold serial dilutions using 7% RMCD to achieve concentrations in the 0.02%–2% *v*/*v* range. For assay calibration, a series of Trolox standard solutions was also prepared in 7% RMCD covering the 6–300 µM concentration range.

The assay was carried out by loading 25 μL of diluted samples/standards and 150-μL of fluorescein solution in a 96-well black polypropylene microplate, followed by incubation at 37 °C for 10 min and the rapid addition of 25 μL of AAPH solution using a 8-channel pipette. Subsequently, fluorescence (excitation: 485 nm, emission: 535 nm) was measured by microplate reader every 2 min for over 2 h and the fluorescence decay curves were used to calculate the area under the curve (AUC). By subtracting the AUC of blanks, the net AUC of the standards and samples was calculated and the ORAC values of the samples were determined using the regression equation between Trolox concentration and net AUC. The ORAC values were calculated across all serial dilutions and the final value of each EO was obtained by averaging the results over the range of dilutions exhibiting steady levels.

The antioxidant capacity of EOs was also determined using the ABTS^•+^ [2,2′-azino-bis(3-ethlybenzthizoline-6-sulphonic acid] radical scavenging assay [65], which is based on both single electron transfer (SET) and hydrogen atom transfer (HAT) reactions (SET/HAT-based). The assay is compatible with both hydrophilic and lipophilic components and thus it measures the total antioxidant capacity in complex samples. For this assay, ABTS^•+^ was prepared by mixing 7 mM ABTS with 2.4 mM potassium persulfate (1:1 *v*/*v*; both solutions prepared in water) and leaving the mixture to react for 24 h in the dark. The EOs were subjected to 2-fold serial dilutions with ethanol to achieve concentrations in the 0.001–2% range. For assay calibration, a series of Trolox standard solutions was also prepared in the concentration range of 6–300 µM. On the day of analysis, the ABTS^•+^ solution was diluted with ethanol to an absorbance of 0.700 at 730 nm. The assay was carried out by loading 10-μL aliquots of diluted samples/standards in a 96-well polystyrene microplate, followed by the rapid addition of 190 μL of diluted ABTS^•+^ using an eight-channel pipette. The microplate was incubated at 28 °C for 60 min, after which the absorbance of samples/standards at 730 nm was recorded using the microplate reader. The calibration curve obtained from Trolox standards was used to calculate the Total Antioxidant Capacity (TAC) across the serial dilutions of each EO, while the final result was obtained by averaging the measurements over the range of dilutions exhibiting steady TAC values. 

The CUPRAC (CUPric Reducing Antioxidant Capacity) technique developed by Özyürek et al. (2008) and Özyürek et al. (2011) was applied with some modifications to suite 96-well microplate format [66,67]. This assay measures total antioxidant capacity based on electron-transfer reactions (ET-based) and it is responsive to both hydrophilic and lipophilic components. Prior to measurements, a 10 mM solution of copper(II) in water (34.1 mg of CuCl_2_·2H_2_O in 20 mL water) and a 7.5 mM neocuproine solution in ethanol (31.2 mg of neocuproine in 20 mL of ethanol) were prepared. A buffer of ammonium acetate buffer 1.0 M, pH 7.0 was also prepared by dissolving 19.27 g NH_4_Ac in 250 mL water, while a 2% solution of methyl-β-cyclodextrin (M-β-CD) was prepared in ethanol. The EOs were initially dissolved in M-β-CD to make 2% (*v*/*v*) solutions and then subjected to 2-fold serial dilutions with the same solvent to achieve concentrations in the 0.01%-2% range. For assay calibration, a series of Trolox standard solutions was also prepared in the concentration range of 6–300 µM. The assay was carried out by mixing 50 μL of copper(II) solution, 50 μL of neocuproine solution, 70 μL of ammonium acetate buffer and 30 μL of diluted sample or standard in a 96-well polystyrene microplate. The microplate was incubated at 25 °C for 60 min, after which the absorbance of samples/standards at 450 nm was recorded against a reagent blank using the microplate reader. The calibration curve obtained from Trolox standards was used to calculate TAC across the serial dilutions of each EO, while the final result was obtained by averaging the measurements over the range of dilutions exhibiting steady TAC values. The results from the different assays are expressed as micromoles of Trolox equivalents per millilitre of oil (TEAC in μmol mL^−1^).

### 3.5. Statistical Analysis

Hierarchical Cluster Analysis (HCA) was performed using OriginPro (version 2016; OriginLab Corp., Northampton, MA, USA) to identify the similarities/differences among the studied EOs with respect to their chemical composition. The concentrations of the most abundant chemical constituents (i.e., 40 compounds showing an abundance of ≥3% in at least one EO) were included in this analysis. The Euclidean distance was applied as dissimilarity measure and the average linkage was employed for hierarchical clustering of EOs.

Principal component analysis (PCA) with standardized data was also applied in order to identify putative relationships between chemical composition, total antioxidant capacity and anti-bacterial activity of EOs. Similar to HCA, the percentage concentrations of the 40 most abundant constituents were used as active variables in PCA. The total antioxidant capacity and anti-bacterial activity of EOs were also projected onto the PCA plot as supplementary variables (i.e., not taken into account for the computation of the components). The PCA was conducted based on Pearson’s correlation matrix by using the XLSTAT software (version 2016; Addinsoft Inc., New York, NY, USA).

## 4. Conclusions

In an attempt to find natural antibiotic alternatives that could substitute synthetic anti-bacterials in fish aquaculture, essential oils from 12 medicinal-aromatic Mediterranean plants were examined in vitro against nine bacterial pathogens commonly implicated in outbreaks across the fish farms of Greece. Analyses showed that all plant extracts exhibit antimicrobial properties, which differ in their effectiveness depending on their chemical composition. Carvacrol, p-cymene and γ-terpinene were considered as the compounds that contribute most to the growth inhibition of the nine fish pathogens. These components were present in the essential oils from *Thymbra capitata*, *Satureja thymbra* and *Origanum vulgare* subsp. *hirtum*. Furthermore, the EOs from these three plants were found to be the most efficient free radical scavengers, according to the results acquired from three different antioxidant capacity assays. In this context, further in vivo tests would show whether these naturally occurring plant products are effective in real fish without side effects. If this proves to be the case, the use of EOs could be applicable both for prophylactic and therapeutic purposes in large-scale fish aquaculture.

## Figures and Tables

**Table 1:** Percentage contribution of the various compound classes in the 13 essential oils under investigation.

Compound Class	Lamiaceae									Apiaceae			Asteraceae
	Pennyroyal ^a^	Pennyroyal ^b^	Lavender	Greek Oregano	Greek Sage	Rosemary	Spanish Oregano	Savory	Lemon Balm	Fennel	Rock Samphire	Wild Carrot	Chamomile
Monoterpene hydrocarbons	1.2	3.4	6.9	16.1	15.6	24.8	47.0	52.2	1.6	0.0	93.9	38.3	0.0
Oxygenated monoterpenes	91.3	90.0	76.5	74.1	69.0	64.6	44.1	36.0	29.6	95.0	4.1	14.8	1.0
Sesquiterpene hydrocarbons	0.0	1.8	4.9	0.0	8.5	3.7	3.4	6.9	53.9	0.0	0.0	30.5	40.1
Oxygenated sesquiterpenes	0.0	0.0	0.0	0.0	0.0	0.0	0.0	0.0	3.7	0.0	0.0	0.0	51.3
Other compounds	2.0	0.0	4.4	0.0	0.0	0.0	1.0	0.0	0.0	0.0	0.0	0.0	4.8
Total identified	94.5	95.2	92.7	90.2	93.1	93.1	95.5	95.1	88.8	95.0	98.0	83.6	97.2

^a^ Pennyroyal oil from Ikaria. ^b^ Pennyroyal oil from Thessaloniki.

**Table 2:** The average IC_50_ values (in μg mL^−1^) of the investigated essential oils and two standard antibiotics (positive controls) against nine bacterial fish pathogens (standard deviation is presented in parentheses).

Material	Bacterial Pathogens ^d^								
	*Aeromonas veronii bv. sobria* (NS2)	*Aeromonas veronii bv. sobria* (NS22)	*Aeromonas veronii bv. sobria* (PDB)	*Aeromonas veronii bv. sobria* (NS13)	*Vibrio anguillarum* (Vak)	*Vibrio harveyi* (VhP1)	*Vibrio alginolyticus* (V1)	*Edwardsiella anguillarum* (EA011113)	*Photobacterium damselae* subsp. *Piscicida* (PdKef)
Pennyroyal ^a^	315 (±17)	298 (±9)	300 (±9)	283 (±24)	127 (±11)	310 (±7)	375 (±4)	193 (±27)	34 (±2)
Pennyroyal ^b^	333 (±13)	348 (±5)	379 (±1)	346 (±9)	152 (±5)	369 (±10)	n.d.^c^	243 (±15)	26 (±4)
Lavender	302 (±64)	311 (±96)	328 (±116)	350 (±85)	124 (±26)	335 (±98)	n.d.^c^	170 (±25)	27 (±2)
Greek oregano	64 (±3)	61 (±1)	61 (±1)	67 (±1)	48 (±2)	59 (±4)	85 (±5)	52 (±3)	27 (±2)
Greek sage	340 (±9)	233 (±12)	309 (±15)	330 (±27)	71 (±4)	393 (±23)	733 (±157)	102 (±10)	32 (±7)
Rosemary	244 (±37)	110 (±10)	180 (±17)	232 (±16)	52 (±6)	162 (±33)	319 (±11)	81 (±2)	30 (±3)
Spanish oregano	69 (±2)	58 (±2)	62 (±0)	63 (±1)	31 (±1)	68 (±2)	88 (±2)	49 (±3)	26 (±1)
Savoury	70 (±6)	58 (±0)	63 (±0)	64 (±8)	31 (±2)	63 (±5)	94 (±5)	52 (±1)	26 (±1)
Lemon balm	360 (±13)	302 (±4)	324 (±2)	274 (±26)	64 (±3)	236 (±10)	338 (±22)	53 (±4)	20 (±1)
Fennel	275 (±11)	330 (±18)	448 (±4)	230 (±15)	160 (±11)	306 (±9)	527 (±39)	223 (±30)	71 (±16)
Wild carrot	185 (±16)	124 (±0)	187 (±2)	143 (±11)	19 (±1)	57 (±12)	138 (±13)	32 (±2)	16 (±0)
Rock samphire	280 (±3)	261 (±9)	360 (±3)	365 (±11)	152 (±17)	223 (±15)	413 (±5)	169 (±5)	46 (±3)
Chamomile	985 (±86)	904 (±23)	966 (±84)	598 (±29)	389 (±31)	632 (±20)	n.d. ^c^	339 (±10)	173 (±56)
Florfenicol ^e^	0.36 ± 0.03	0.30 ± 0.01	0.37 ± 0.01	0.26 ± 0.01	0.46 ± 0.01	0.67 ± 0.06	0.53 ± 0.04	0.24 ± 0.02	0.33 ± 0.03
Oxytetracycline ^e^	0.061 ± 0.002	0.045 ± 0.001	0.072 ± 0.002	0.057 ± 0.005	0.046 ± 0.003	0.093 ± 0.002	0.082 ± 0.005	0.115 ± 0.001	0.071 ± 0.007

^a^ Pennyroyal oil from Ikaria. ^b^ Pennyroyal oil from Thessaloniki ^c^ n.d. denotes not determined. ^d^ Species names are given together with respective codes in parentheses. ^e^ Standard antibiotics used as positive controls.

**Table 3:** Comparison of the antioxidant capacity of the 13 essential oils investigated in the present study. Presented results correspond to Trolox equivalent antioxidant capacity (in μmol mL^−1^), as measured by CUPRAC (cupric reducing antioxidant capacity), ABTS (2,2′-azino-bis(3-ethlybenzthizoline-6-sulphonic acid radical scavenging) and ORAC (oxygen radical absorbance capacity) assays.

Essential Oil	CUPRAC	ABTS	ORAC
Pennyroyal ^a^	44 (±5)	154 (±15)	2818 (±512)
Pennyroyal ^b^	37 (±1)	32 (±2)	777 (±43)
Lavender	32 (±0.1)	5.6 (±1)	1698 (±100)
Greek oregano	1441 (±271)	5879 (±325)	4147 (±578)
Greek sage	70 (±0.1)	4.1 (±0.1)	747 (±88)
Rosemary	25 (±3)	2.7 (±0.4)	854 (±120)
Spanish oregano	2733 (±264)	5072 (±253)	5933 (±614)
Savoury	931 (±136)	2591 (±14)	4025 (±203)
Lemon balm	98 (±7)	68 (±5)	2488 (±171)
Fennel	62 (±1)	38 (±1)	2389 (±162)
Wild carrot	44 (±4)	13 (±2)	2560 (±130)
Rock samphire	184 (±3)	30 (±4)	1283 (±74)
Chamomile	13 (±2)	7.4 (±0.2)	702 (±125)

^a^ Pennyroyal oil from Ikaria. ^b^ Pennyroyal oil from Thessaloniki.

**Table 4:** Plant species used for the extraction of the 13 essential oils under investigation.

Common Name	Scientific Name (Family)	Cultivation Area	Abbreviation
Pennyroyal	*Mentha pulegium* (Lamiaceae)	Ikaria	MP-1
Pennyroyal	*Mentha pulegium* (Lamiaceae)	Thessaloniki	MP-2
Lavender	*Lavandula angustifolia* ‘hemus’ (Lamiaceae)	Grevena	LA
Greek oregano	*Origanum vulgare* subsp. *hirtum* (Lamiaceae)	Ptolemaida	OVH
Greek sage	*Salvia fruticosa* (Lamiaceae)	Ikaria	SF
Rosemary	*Rosmarinum officinalis* (Lamiaceae)	Ptolemaida	RO
Spanish oregano	*Thymbra capitata* (Lamiaceae)	Ikaria	TC
Savoury	*Satureja thymbra* (Lamiaceae)	Ikaria	ST
Lemon balm	*Melissa officinalis* (Lamiaceae)	Ptolemaida	MO
Fennel	*Foeniculum vulgare* (Apiaceae)	Thessaloniki	FV
Wild carrot	*Daucus carota* (Apiaceae)	Ikaria *	DC
Rock samphire	*Crithmum maritimum* (Apiaceae)	Thessaloniki	CM
Chamomile	*Matricaria chamomilla* (Asteraceae)	Thessaloniki	MC

* Wild-harvested.

**Table 5:** Bacterial fish pathogens isolated from Greek aquaculture facilities.

Bacterial Species	Strain Code	Aquaculture Location	Fish Species (Common Name)	Fish Species (Scientific Name)	Culture Medium
*Aeromonas veronii bv. sobria*	NS2	Argolis	European seabass	*Dicentrarchus labrax*	BHI ^a^
*Aeromonas veronii bv. sobria*	NS22	Peloponnese	European seabass	*Dicentrarchus labrax*	BHI ^a^
*Aeromonas veronii bv. sobria*	PDB	Argolis	European seabass	*Dicentrarchus labrax*	BHI ^a^
*Aeromonas veronii bv. sobria*	NS13	Argolis	European seabass	*Dicentrarchus labrax*	BHI ^a^
*Vibrio anguillarum*	Vak	Cephalonia	European seabass	*Dicentrarchus labrax*	TSA ^b^
*Vibrio harveyi*	VhP1	Kalymnos	European seabass	*Dicentrarchus labrax*	TSA ^b^
*Vibrio alginolyticus*	V1	Crete	gilthead seabream	*Sparus aurata*	TSA ^b^
*Edwardsiella anguillarum*	EA011113	Saronikos	sharpsnout seabream	*Diplodus puntazzo*	LB ^c^
*Photobacterium damselae* subsp. *piscicida*	PdKef	Lefkada	common seabream	*Pagrus pagrus*	LB ^c^

^a^ Brain Heart Infusion (BHI) growth medium with 0.5% NaCl. ^b^ Tryptic Soy Agar (TSA) growth medium. ^c^ Lysogeny Broth (LB) growth medium with 2.0% NaCl.

## Supplementary Materials

The following are available online at https://www.mdpi.com/1420-3049/25/1/148/s1, Tables S1 and S2; Figure S1.

## References

[1] Pridgeon J.W., Klesius P.H. (2012). Major bacterial diseases in aquaculture and their vaccine development. Cab Rev..

[2] Rigos G., Troisi G.M. (2005). Anti-bacterial agents in Mediterranean finfish farming: A synopsis of drug pharmacokinetics in important euryhaline fish species and possible environmental implications. Rev. Fish Biol. Fish..

[3] Samanidou V.F., Evaggelopoulou E.N. (2007). Analytical strategies to determine antibiotic residues in fish. J. Sep. Sci..

[4] Romero J., Feijoó C.G., Navarrete P. (2012). Antibiotics in aquaculture—Use, abuse and alternatives. Health Environ. Aquac..

[5] Navarrete P., Mardones P., Opazo R., Espejo R., Romero J. (2008). Oxytetracycline treatment reduces bacterial diversity of intestinal microbiota of Atlantic salmon. J. Aquat. Anim. Health.

[6] Romero J., Ringø E., Merrifield D.L. (2014). The gut microbiota of fish. Aquac. Nutr..

[7] Cabello F.C., Godfrey H.P., Buschmann A.H., Dölz H.J. (2016). Aquaculture as yet another environmental gateway to the development and globalisation of antimicrobial resistance. Lancet Infect. Dis..

[8] Watts J.E.M., Schreier H.J., Lanska L., Hale M.S. (2017). The rising tide of antimicrobial resistance in aquaculture: Sources, sinks and solutions. Mar. Drugs.

[9] Sommerset I., Krossøy B., Biering E., Frost P. (2005). Vaccines for fish in aquaculture. Expert Rev. Vaccines.

[10] Mulero I., Sepulcre M.P., Fuentes I., García-Alcázar A., Meseguer J., García-Ayala A., Mulero V. (2008). Vaccination of larvae of the bony fish gilthead seabream reveals a lack of correlation between lymphocyte development and adaptive immunocompetence. Mol. Immunol..

[11] Edris A.E. (2007). Pharmaceutical and therapeutic potentials of essential oils and their individual volatile constituents: A review. Phyther. Res..

[12] Orchard A., van Vuuren S. (2017). Commercial essential oils as potential antimicrobials to treat skin diseases. Evid.-Based Complement. Altern. Med..

[13] Elshafie H.S., Camele I. (2017). An overview of the biological effects of some Mediterranean essential oils on human health. Biomed. Res. Int..

[14] Sutili F.J., Gatlin D.M.I., Heinzmann B.M., Baldisserotto B. (2017). Plant essential oils as fish diet additives: Benefits on fish health and stability in feed. Rev. Aquac..

[15] Dhifi W., Bellili S., Jazi S., Bahloul N., Mnif W. (2016). Essential oils’ chemical characterization and investigation of some biological activities: A critical review. Medicines.

[16] Hassoun A., Çoban Ö.E. (2017). Essential oils for antimicrobial and antioxidant applications in fish and other seafood products. Trends Food Sci. Technol..

[17] Harikrishnan R., Balasundaram C., Heo M.-S. (2011). Impact of plant products on innate and adaptive immune system of cultured finfish and shellfish. Aquaculture.

[18] Reverter M., Bontemps N., Lecchini D., Banaigs B., Sasal P. (2014). Use of plant extracts in fish aquaculture as an alternative to chemotherapy: Current status and future perspectives. Aquaculture.

[19] Awad E., Awaad A. (2017). Role of medicinal plants on growth performance and immune status in fish. Fish Shellfish Immunol..

[20] Nazzaro F., Fratianni F., De Martino L., Coppola R., De Feo V. (2013). Effect of essential oils on pathogenic bacteria. Pharmaceuticals.

[21] da Cunha J.A., Heinzmann B.M., Baldisserotto B. (2018). The effects of essential oils and their major compounds on fish bacterial pathogens—A review. J. Appl. Microbiol..

[22] Misharina T.A., Terenina M.B., Krikunova N.I. (2009). Antioxidant properties of essential oils. Appl. Biochem. Microbiol..

[23] Elabd H., Wang H.P., Shaheen A., Yao H., Abbass A. (2017). Anti-oxidative effects of some dietary supplements on Yellow perch (*Perca flavescens*) exposed to different physical stressors. Aquac. Rep..

[24] Milina R., Mustafa Z., Stanev S., Zvezdova D., Stoeva S. (2012). Headspace gas chromatographic analysis of Bulgarian *Lavandula Angustifolia* mill Herbs. I. optimization of the analysis conditions. Нaучни Трудoве Нa Русенския Университет (Sci. Works Univ. (Bulgarian)).

[25] Zagorcheva T., Stanevs S., Rusanov K., Atanassov I. (2013). Comparitive GC/MS analysis of lavender (*Lavandula angustifolaila* Mill.) inflorescence and essential oil volatiles. J. Agric. Sci. Technol..

[26] Stanev S., Zagorcheva T., Atanassov I. (2016). Lavender cultivation in Bulgaria—21st century developments, breeding challenges and opportunities. Bulg. J. Agric. Sci..

[27] Salas J.B., Téllez T.R., Alonso M.J.P., Pardo F.M.V., Capdevila M., de los Á.C., Rodríguez C.G. (2010). Chemical composition and antioxidant activity of the essential oil of *Thymbra capitata* (L.) Cav. in Spain. Acta Bot. Gall..

[28] Elmi A., Ventrella D., Barone F., Filippini G., Benvenuti S., Pisi A., Scozzoli M., Bacci M.L. (2017). *Thymbra capitata* (L.) cav. and *Rosmarinus*
*officinalis* (L.) essential oils: In vitro effects and toxicity on swine spermatozoa. Molecules.

[29] Delgado-Adámez J., Garrido M., Bote M.E., Fuentes-Pérez M.C., Espino J., Martín-Vertedor D. (2017). Chemical composition and bioactivity of essential oils from flower and fruit of *Thymbra capitata* and *Thymus* species. J. Food Sci. Technol..

[30] Vokou D., Kokkini S., Bessiere J.-M. (1993). Geographic variation of Greek oregano (*Origanum vulgare* ssp. *hirtum*) essential oils. Biochem. Syst. Ecol..

[31] Adam K., Sivropoulou A., Kokkini S., Lanaras T., Arsenakis M. (1998). Antifungal activities of *Origanum vulgare* subsp. *hirtum, Mentha spicata, Lavandula angustifolia*, and *Salvia fruticosa* essential oils against human pathogenic fungi. J. Agric. Food Chem..

[32] Kokkini S., Karousou R., Hanlidou E., Lanaras T. (2004). Essential oil composition of Greek (*Origanum vulgare* ssp. hirtum) and Turkish (*O. onites*) oregano: A tool for their distinction. J. Essent. Oil Res..

[33] El Beyrouthy M., Arnold-Apostolides N., Cazier F., Najm S., Abou Jaoudeh C., Labaki M., Dhifi W., Abou Kais A. (2013). Chemical composition of the essential oil of aerial parts of *Satureja Thymbra*, L. growing wild in Lebanon. Acta Hortic..

[34] Piras A., Cocco V., Falconieri D., Porcedda S., Marongiu B., Maxia A., Frau M.A., Gonçalves M.J., Cavaleiro C., Salgueiro L. (2011). Isolation of the volatile oil from *Satureja thymbra* by supercritical carbon dioxide extraction: Chemical composition and biological activity. Nat. Prod. Commun..

[35] Karousou R., Koureas D.N., Kokkini S. (2005). Essential oil composition is related to the natural habitats: *Coridothymus capitatus* and *Satureja thymbra* in NATURA 2000 sites of Crete. Phytochemistry.

[36] Telci I., Demirtas I., Sahin A. (2009). Variation in plant properties and essential oil composition of sweet fennel (*Foeniculum vulgare* Mill.) fruits during stages of maturity. Ind. Crop. Prod..

[37] Diao W.-R., Hu Q.-P., Zhang H., Xu J.-G. (2014). Chemical composition, anti-bacterial activity and mechanism of action of essential oil from seeds of fennel (*Foeniculum vulgare* Mill.). Food Control.

[38] Zoubiri S., Baaliouamer A., Seba N., Chamouni N. (2014). Chemical composition and larvicidal activity of Algerian *Foeniculum vulgare* seed essential oil. Arab. J. Chem..

[39] Shahmohamadi R., Sariri R., Rasa M., Ghafoori H., Aghamali M., Nasuti S., Tahery M. (2011). Chemical composition and antimicrobial activity of flowering aerial parts *Mentha pulegium* from Gilan. Pharmacologyonline.

[40] Bouyahya A., Et-Touys A., Bakri Y., Talbaui A., Fellah H., Abrini J., Dakka N. (2017). Chemical composition of *Mentha pulegium* and *Rosmarinus officinalis* essential oils and their antileishmanial, anti-bacterial and antioxidant activities. Microb. Pathog..

[41] Zanjani M.A.K., Mohammadi N., Zojaji M., Bakhoda H. (2015). Chemical composition of the essential oil of *Mentha pulegium* L. and its antimicrobial activity on *Proteus mirabilis*, *Bacillus subtilis* and *Zygosaccharomyces rouxii*. J. Food Biosci. Technol..

[42] Calo J.R., Crandall P.G., O’Bryan C.A., Ricke S.C. (2015). Essential oils as antimicrobials in food systems—A review. Food Control.

[43] Tu X.-F., Hu F., Thakur K., Li X.-L., Zhang Y.-S., Wei Z.-J. (2018). Comparison of anti-bacterial effects and fumigant toxicity of essential oils extracted from different plants. Ind. Crop. Prod..

[44] Helander I.M., Alakomi H.-L., Latva-Kala K., Mattila-Sandholm T., Pol I., Smid E.J., Gorris L.G.M., von Wright A. (1998). Characterization of the action of selected essential oil components on Gram-negative bacteria. J. Agric. Food Chem..

[45] La Storia A., Ercolini D., Marinello F., Di Pasqua R., Villani F., Mauriello G. (2011). Atomic force microscopy analysis shows surface structure changes in carvacrol-treated bacterial cells. Res. Microbiol..

[46] Dorman H.J.D., Deans S.G. (2000). Antimicrobial agents from plants: Anti-bacterial activity of plant volatile oils. J. Appl. Microbiol..

[47] Hyldgaard M., Mygind T., Meyer R.L. (2012). Essential oils in food preservation: Mode of action, synergies, and interactions with food matrix components. Front. Microbiol..

[48] Ultee A., Slump R.A., Steging G., Smid E.J. (2000). Antimicrobial activity of carvacrol toward *Bacillus cereus* on rice. J. Food Prot..

[49] Ghorbanpour M., Hadian J., Hatami M., Salehi-Arjomand H., Aliahmadi A. (2016). Comparison of chemical compounds and antioxidant and anti-bacterial properties of various *Satureja* species growing wild in Iran. J. Med. Plants.

[50] Burt S. (2004). Essential oils: Their anti-bacterial properties and potential applications in foods—A review. Int. J. Food Microbiol..

[51] Ran C., Hu J., Liu W., Liu Z., He S., Dan B.C.T., Diem N.N., Ooi E.L., Zhou Z. (2016). Thymol and carvacrol affect hybrid tilapia through the combination of direct stimulation and an intestinal microbiota-mediated effect: Insights from a germ-free zebrafish model. J. Nutr..

[52] Polednik K.M., Koch A.C., Felzien L.K. (2018). Effects of essential oil from *Thymus vulgaris* on viability and inflammation in zebrafish embryos. Zebrafish.

[53] Volpatti D., Chiara B., Tulli F., Marco G. (2013). Growth parameters, innate immune response and resistance to *Listonella (Vibrio) anguillarum* of *Dicentrarchus labrax* fed carvacrol supplemented diets. Aquac. Res..

[54] Ruberto G., Baratta M.T. (2000). Antioxidant activity of selected essential oil components in two lipid model systems. Food Chem..

[55] de Oliveira T.M., de Carvalho R.B.F., da Costa I.H.F., de Oliveira G.A.L., de Souza A.A., de Lima S.G., de Freitas R.M. (2015). Evaluation of p-cymene, a natural antioxidant. Pharm. Biol..

[56] Amorati R., Foti M.C., Valgimigli L. (2013). Antioxidant activity of essential oils: A critical review. J. Agric. Food Chem..

[57] van Den Dool H., Kratz P.D. (1963). A generalization of the retention index system including linear tempera¬ture programmed gas-liquid partition chromatography. J. Chromatogr. A.

[58] Massada Y. (1976). Analysis of Essential Oils by Gas Chromatography and Mass Spectrometry.

[59] Robert P., Adams R.P. (2007). Identification of Essential Oil Components by Gas Chromatography/Mass Spectrometry.

[60] Smyrli M., Prapas A., Rigos G., Kokkari C., Pavlidis M., Katharios P. (2017). *Aeromonas veronii* infection associated with high morbidity and mortality in farmed European seabass *Dicentrarchus labrax* in the Aegean Sea, Greece. Fish Pathol..

[61] Katharios P., Kokkari C., Dourala N., Smyrli M. (2015). First report of Edwardsiellosis in cage-cultured sharpsnout sea bream, *Diplodus puntazzo* from the Mediterranean. BMC Vet. Res..

[62] Katharios P., Kalatzis P.G., Kokkari C., Pavlidis M., Wang Q. (2019). Characterization of a highly virulent *Edwardsiella anguillarum* strain isolated from Greek aquaculture, and a spontaneously induced prophage therein. Front. Microbiol..

[63] Castillo D., D’Alvise P., Kalatzis P.G., Kokkari C., Middelboe M., Gram L., Liu S., Katharios P. (2015). Draft genome sequences of *Vibrio alginolyticus* strains V1 and V2, opportunistic marine pathogens. Genome Announc..

[64] Mandalakis M., Gavriilidou A., Polymenakou P.N., Christakis C.A., Nomikou P., Medvecký M., Kilias S.P., Kentouri M., Kotoulas G., Magoulas A. (2019). Microbial strains isolated from CO_2_-venting Kolumbo submarine volcano show enhanced co-tolerance to acidity and antibiotics. Mar. Env. Res..

[65] Re R., Pellegrini N., Proteggente A., Pannala A., Yang M., Rice-Evans C. (1999). Antioxidant activity applying an improved ABTS radical cation decolorization assay. Free Radic. Biol. Med..

[66] Özyürek M., Bektaşoǧlu B., Güçlü K., Güngör N., Apak R. (2008). Simultaneous total antioxidant capacity assay of lipophilic and hydrophilic antioxidants in the same acetone-water solution containing 2% methyl-β-cyclodextrin using the cupric reducing antioxidant capacity (CUPRAC) method. Anal. Chim. Acta.

[67] Özyürek M., Güçlü K., Tütem E., Sözgen-Başkan K., Erçaǧ E., Çelik S.E., Baki S., Yildiz L., Karaman Ş., Apak R. (2011). A comprehensive review of CUPRAC methodology. Anal. Methods.

