# Supplementary material for: Comparative Evaluation of Essential Oils from Medicinal-Aromatic Plants of Greece: Chemical Composition, Antioxidant Capacity and Antimicrobial Activity against Bacterial Fish Pathogens
Source: Molecules. 2019 Dec 30;25(1):148. doi: 10.3390/molecules25010148 (PMC6982863; doi:10.3390/molecules25010148)
Supplement: Supplementary file 1 [file molecules-25-00148-s001.pdf]

Supplementary Material for the *Special Issue of* **Molecules**

"Essential Oils as Antimicrobial and Anti-infectious Agents II"

Comparative evaluation of essential oils from medicinal-aromatic plants of Greece: Chemical composition, antioxidant capacity and antimicrobial activity against bacterial fish pathogens

Thekla I. Anastasiou[1], Manolis Mandalakis[1,*], Nikos Krigas[2], Thomas Vézignol[1], Diamanto Lazari[3], Pantelis Katharios[1], Thanos Dailianis[1], Efthimia Antonopoulou[4]

[1] Institute of Marine biology, Biotechnology and Aquaculture, Hellenic Centre of Marine Research, 71500 Heraklion, Greece

[2] Institute of Plant Breeding and Genetic Resources, Hellenic Agricultural Organization Demeter, P.O. Box 60458, 57001 Thermi, Thessaloniki, Greece

[3] Laboratory of Pharmacognosy, School of Pharmacy, Faculty of Health Sciences, Aristotle University of Thessaloniki, 54124 Thessaloniki, Greece

[4] Laboratory of Animal Physiology, Department of Zoology, School of Biology, Faculty of Sciences, Aristotle University of Thessaloniki, 54124, Greece

[*] Corresponding author, E-mail address: mandalakis@hcmr.gr, phone: +30-2810-337855, fax: +30-2810-337822 (M. Mandalakis).

Table S1. Concentrations of individual chemical compounds detected in the 13 essential oils of the Mediterranean medicinal-aromatic plants under investigation.

		Lamiaceae									Apiaceae			Asteraceae				
**No**	**Compounds[a]**	**Pennyroyal[b]**	**Pennyroyal[c]**	**Lavender**	**Greek oregano**	**Greek sage**	**Rosemary**	**Spanish oregano**	**Savoury**	**Lemon balm**	**Fennel**	**Rock samphire**	**Wild carrot**	**Chamomile**	**AI[d]**	**AI[g]**	**Identification[e]**	**Classification[f]**
1	α-Thujene							3.5	2.5						924	924	AI, MS	MH
2	α-Pinene					2.9	8.8	1.6	1.8			7.6	20.5		932	932	AI, MS, Co-GC	MH
3	Camphene					3.6	4						1.1		947	946	AI, MS	MH
4	Sabinene											2.9	2.3		969	969	AI, MS	MH
5	β-Pinene					5.4	7.6		1						974	974	AI, MS, Co-GC	MH
6	1-Octen-3-ol							1							981	974	AI, MS	others
7	3-Octanone			2.1											988	979	AI, MS	others
8	Myrcene			1.2	1.2	3.7	1.1	4.6	2.9				6.9		990	988	AI, MS, Co-GC	MH
9	3-Octanol	2													997	988	AI, MS	others
10	α-Terpinene							3.9	3.5						1014	1014	AI, MS	MH
11	p-Cymene				9.6		1.4	11.9	6.5			6.4			1020	1020	AI, MS, Co-GC	MH
12	Sylvestrene							1							1024	1025	AI, MS	MH
13	Limonene	1.2	3.4				1.9					53.3	7.5		1024	1024	AI, MS, Co-GC	MH
14	Eucalyptol					53.2	45								1026	1026	AI, MS, Co-GC	OM
15	*cis*-Ocimene			2.8								2.3			1032	1032	AI, MS	MH
16	*trans*-Ocimene			2.9						1.6					1044	1044	AI, MS	MH
17	γ-Terpinene				5.3			20.5	34			21.4			1054	1054	AI, MS, Co-GC	MH
18	Artemisia ketone													1	1056	1056	AI, MS	OM
19	Linalool			39.1				1.1	1.4						1099	1095	AI, MS, Co-GC	OM
20	α-Thujone					2.2									1102	1101	AI, MS, Co-GC	OM
21	1-Octen-3-yl acetate			1.3											1110	1110	AI, MS	others
22	Hexyl acetate			1											1112	1112	AI, MS	others
23	β-Thujone					2.5									1113	1112	AI, MS, Co-GC	OM
24	Camphor					8.1	11.5								1141	1141	AI, MS, Co-GC	OM
25	Citronellal									10.2					1148	1148	AI, MS	OM

26	Menthone	3.5	2.8												1148	1148	AI, MS	OM
27	Isomenthone	5.2													1162	1158	AI, MS	OM
28	Borneol						4.3								1163	1165	AI, MS, Co-GC	OM
29	Terpinen-4-ol			2.9				1							1174	1174	AI, MS, Co-GC	OM
30	α-Terpineol						1.9								1186	1186	AI, MS, Co-GC	OM
31	Citronellol									1.8					1223	1223	AI, MS	OM
32	Thymol methyl ether											4.1			1232	1232	AI, MS	OM
33	Pulegone	47.6	87.2												1233	1233	AI, MS	OM
34	Neral									6.5					1235	1235	AI, MS	OM
35	Carvacrol methyl ether								1.8						1246	1241	AI, MS	OM
36	Piperitone	2													1249	1249	AI, MS	OM
37	Linalool acetate			31.5											1254	1254	AI, MS	OM
38	Methyl citronellate									2.3					1257	1257	AI, MS	OM
39	Geranial									8.8					1264	1264	AI, MS	OM
40	*trans*-Anethole										95				1282	1282	AI, MS	OM
41	Bornyl acetate					3	1.9								1287	1287	AI, MS, Co-GC	OM
42	Lavandulyl acetate			3											1288	1288	AI, MS	OM
43	Thymol				2.1										1289	1289	AI, MS, Co-GC	OM
44	Carvacrol				72			42	32.8						1301	1298	AI, MS	OM
45	α-longipinene												5.2		1346	1350	AI, MS	SH
46	Piperitenone	33													1347	1340	AI, MS	OM
47	α-Copaene									2.4					1374	1374	AI, MS	SH
48	β-Bourbonene									1.5					1387	1387	AI, MS	SH
49	β-Caryophyllene			3.1		7.5	3.7	3.4	6.9	27.7					1417	1417	AI, MS, Co-GC	SH
50	γ-Elemene													6.1	1434	1434	AI, MS	SH
51	α-Caryophyllene					1				2.2					1446	1452	AI, MS, Co-GC	SH
52	*trans*-β-Farnesene			1.8						1.2				12.6	1454	1454	AI, MS	SH
53	γ-Muurolene									12.6					1478	1478	AI, MS	SH
54	α-Cedrene												3.7		1479	1481	AI, MS	SH
55	Germacrene D		1.8											3.8	1484	1484	AI, MS	SH
56	Isoeugenol methyl ether												14.8		1499	1500	AI, MS	OM

57	α-(*E*,*E*)-Farnesene	1.8		1.3	1505	1505	AI, MS	SH
58	γ-Cadinene	1.6			1513	1513	AI, MS	SH
59	δ-Cadinene	2.9			1518	1522	AI, MS	SH
60	Caryophyllene oxide	2.7			1578	1582	AI, MS, Co-GC	OS
61	β-Himachalene		21.6		1647	1652	AI, MS	SH
62	α-Cadinol	1			1652	1652	AI, MS	OS
63	α-Bisabolol oxide B			23.3	1656	1656	AI, MS	OS
64	α-Bisabolone oxide A			16.3	1684	1684	AI, MS	OS
65	Chamazulene			16.3	1730	1730	AI, MS	SH
66	α-Bisabolol oxide A			11.7	1748	1748	AI, MS	OS
67	(Z)-Spiroether			4.8	1879	1879	AI, MS	others

[a] Compounds are listed in order of elution from an HP-5 MS capillary column
[b] Pennyroyal oil from Ikaria
[c] Pennyroyal oil from Thessaloniki
[d] Arithmetic indices (AI) determined on a HP-5 MS capillary column using a homologous series of n-alkanes (C9-C25)
[e] Identification method: AI=Arithmetic Index, MS=mass spectrum, Co-GC=Coinjection with authentic compound
[f] MH denotes monoterpene hydrocarbons, OM denotes oxygenated monoterpenes, SH denotes sesquiterpene hydrocarbons and OS denotes oxygenated sesquiterpenes
[g] Arithmetic indices (AI) from literature data

Table S2. Comparison of the major components detected in each essential oil of the studied Mediterranean medicinal-aromatic plants with those reported in the literature.

Common plant name	Compound	Literature
Pennyroyal	pulegone (69.22%); menthone (18.98%)	[1]
	pulegone (40.98%); menthone (21.164%)	[2]
	pulegone (19.89%); eucalyptol (19.38%); piperitenone (15.14%)	[3]
	pulegone (47.6%-87.2%); piperitenone (n.d.-33%)	*present study*
Lavender	linalool acetate (37.63%); linalool (36.26%)	[4]
	linalool acetate (30.99%); linalool (23.13%)	[5]
	linalool (30.5%-39.8%); linalool acetate (26.7%-37.9%)	[6]
	linalool (39.1%); linalool acetate (31.5%)	*present study*
Greek oregano	carvacrol (2.3%-93.8%); thymol (0.2%-90.2%); γ-terpinene (0.1%-16.4%); p-cymene (2.2%-15.8%)	[7]
	thymol (45.22%); carvacrol (33.05%); p-cymene (7.35%)	[8]
	carvacrol (1.7%-69.6%); thymol (0.2%-42.8%); p-cymene (17.3%-51.3%);	[9]
	carvacrol (72%); p-cymene (9.6%); γ-terpinene (5.3%)	*present study*
Greek sage	eucalyptol (46.0%-58.9%); camphor (0.7%-5.8%)	[10]
	eucalyptol (78%); α-thujone (4.2%)	[11]
	eucalyptol (43.10%); camphor (18.34%)	[8]
	eucalyptol (53.2%); camphor (8.1%)	*present study*
Rosemary	eucalyptol (48.3%-58.7%); borneol (8.8%-10.4%); a-pinene (7.9%-9.9%)	[10]
	eucalyptol (88.9%); a-pinene (2.7%); camphor (2.4%)	[11]
	a-pinene (23.55%); camphor (22.03%); eucalyptol (21.36%)	[12]
	a-pinene (13.7%-24.6%); bornyl acetate (11.3%-17.0%); verbenone (4.4%-24.9%)	[13]
	eucalyptol (45.0%); camphor (11.5%); a-pinene (8.8%)	*present study*
Spanish oregano	carvacrol (66.2%-75.2%); γ-terpinene (3.4%-11.2%); p-cymene (7.3%-8.4%)	[14]
	carvacrol (65.2%); p-cymene (12.28%); γ-terpinene (5.62%)	[12]
	carvacrol (74.27%-75.51%); p-cymene (7.29%-9.10%); γ-terpinene (4.14%-4.83%)	[15]
	carvacrol (42%); γ-terpinene (20.5%); p-cymene (11.9%)	*present study*
Savoury	γ-terpinene (34.06%); carvacrol (23.07%); thymol (18.82%); p-cymene (7.58%)	[16]

	thymol (57.3%-64%); γ-terpinene (7.2%-9.8%); p-cymene (6.3%-9.8%)	[17]
	carvacrol (5.2%-66.5%); thymol (0.1%-65.6%); γ-terpinene (4.4%-22.6%); p-cymene (5.5%-14.8%)	[18]
	γ-terpinene (34%); carvacrol (32.8%); p-cymene (6.5%)	*present study*
Lemon balm	geranial (44.2%); neral (30.2%); citronellal (6.3%)	[19]
	(E)-citral (37.2%); neral (23.9%); citronellal (20.3%)	[20]
	β-caryophyllene (27.7%); γ-muurolene (12.6%); citronellal (10.2%)	*present study*
Fennel	trans-anethole (68.53%); chavicol methyl ether (10.42%)	[21]
	trans-anethole (70.13%-76.37%); fenchone (10.04%-14.17%)	[22]
	trans-anethole (81.63%-87.85%); chavicol methyl ether (4.19%-5.53%)	[23]
	trans-anethole (95%)	*present study*
Wild carrot	α-pinene (7.05%-51.23%); sabinene (2.68%-36.39%); α-muurolene (0.24%-10.97%)	[24]
	carotol (66.78%); daucene (8.74%)	[25]
	isoeugenol methyl ether (33.0%); α-pinene (24.9%); elemicin (11.4%)	[26]
	β-himachalene (21.6%); α-pinene (20.5%); isoeugenol methyl ether (14.8%)	*present study*
Rock samphire	limonene (57.5%-74.2%); sabinene (8.1%-13.4%); γ-terpinene (4.6%-13.8%)	[27]
	γ-terpinene (22.54%-43.29%); methyl thymyl ether (20.13%-34.29%); dillapiole (2.39%-41.35%); p-cymene (4.83%-22.08%)	[28]
	sabinene (26.9%); limonene (24.2%); γ-terpinene (19.3%)	[29]
	limonene (53.3%); γ-terpinene (21.4%); a-pinene (7.6%)	*present study*
Chamomile	α-bisabolol oxide B (15.58%-35.63%); α-bisabolol oxide A (17.46%-35.38%); chamazulene (15.83%-19.27%); 8-isobutyryloxy isobornyl isobutyrate (11.15%-14.03%)	[30]
	trans-β-farnesene (29.8%); (E,E)-α-farnesene (9.3%); α-bisabolol oxide A (7.0%); chamazulene (6.4%)	[31]
	trans-β-farnesene (24.19%); guaiazulene (10.57%); α-bisabolol oxide A (10.21%); α-farnesene (8.70%)	[32]
	α-bisabolol oxide B (23.3%); α-bisabolone oxide A (16.3%); chamazulene (16.3%); trans-β-farnesene (12.6%); α-bisabolol oxide A (11.7%)	*present study*

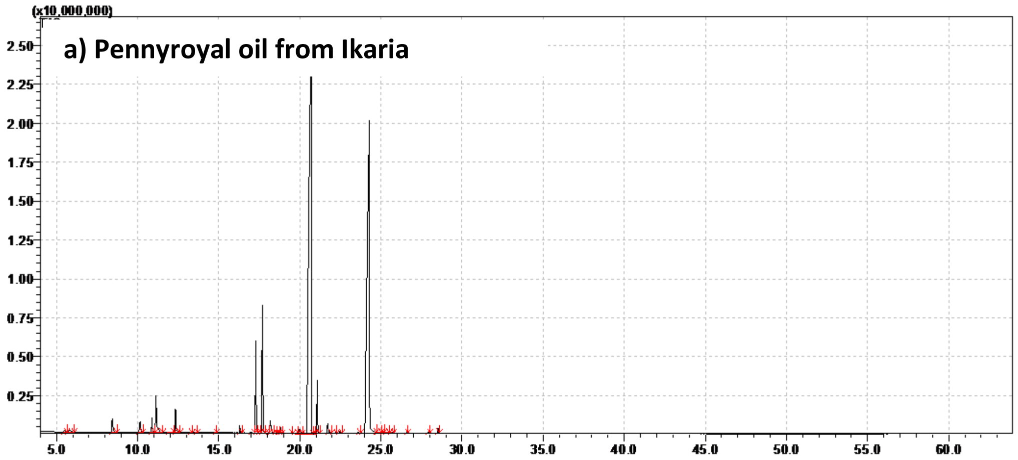
(x10,000,000)
a) Pennyroyal oil from Ikaria
2.50
2.25
2.00
1.75
1.50
1.25
1.00
0.75
0.50
0.25
5.0 10.0 15.0 20.0 25.0 30.0 35.0 40.0 45.0 50.0 55.0 60.0

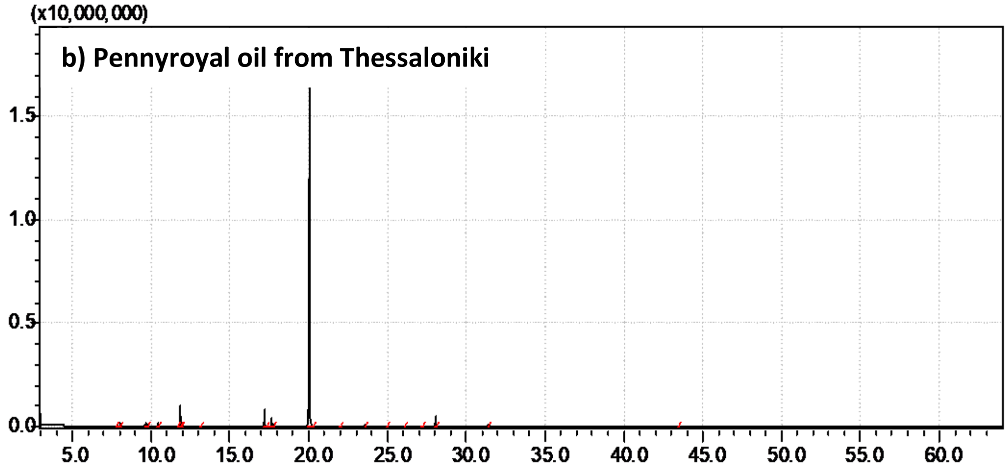
(x10,000,000)
b) Pennyroyal oil from Thessaloniki
1.5
1.0
0.5
0.0
5.0 10.0 15.0 20.0 25.0 30.0 35.0 40.0 45.0 50.0 55.0 60.0

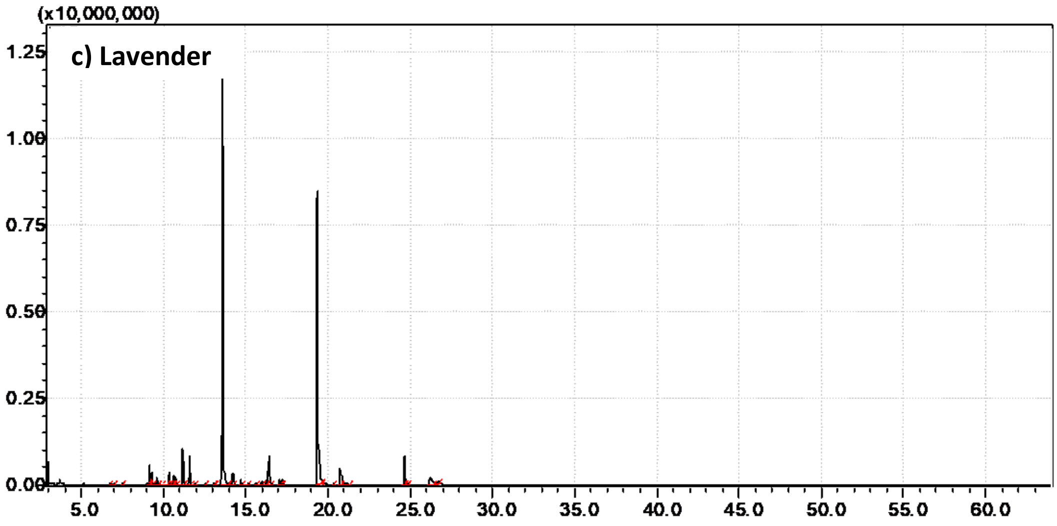
(x10,000,000)
c) Lavender
1.25
1.00
0.75
0.50
0.25
0.00
5.0 10.0 15.0 20.0 25.0 30.0 35.0 40.0 45.0 50.0 55.0 60.0

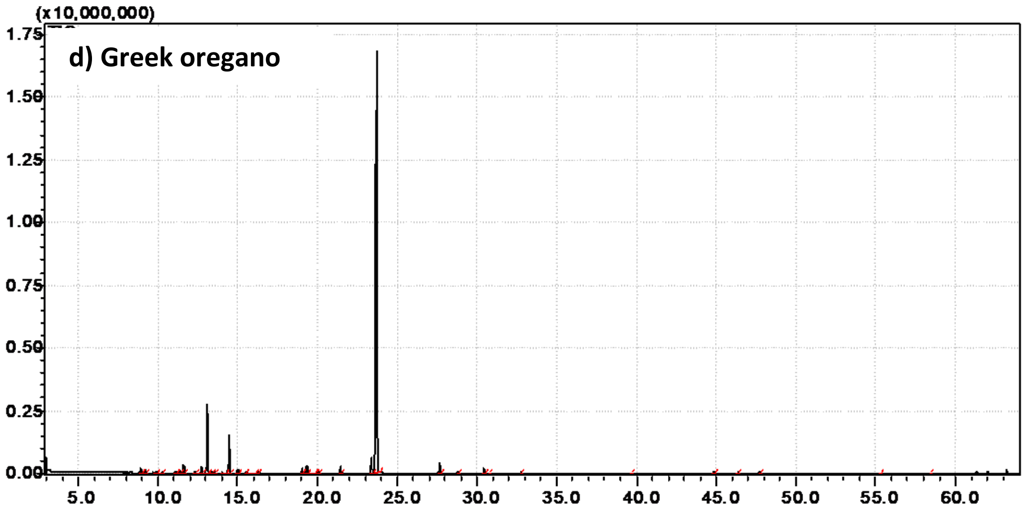
(x10,000,000)
d) Greek oregano
1.75
1.50
1.25
1.00
0.75
0.50
0.25
0.00
5.0
10.0
15.0
20.0
25.0
30.0
35.0
40.0
45.0
50.0
55.0
60.0

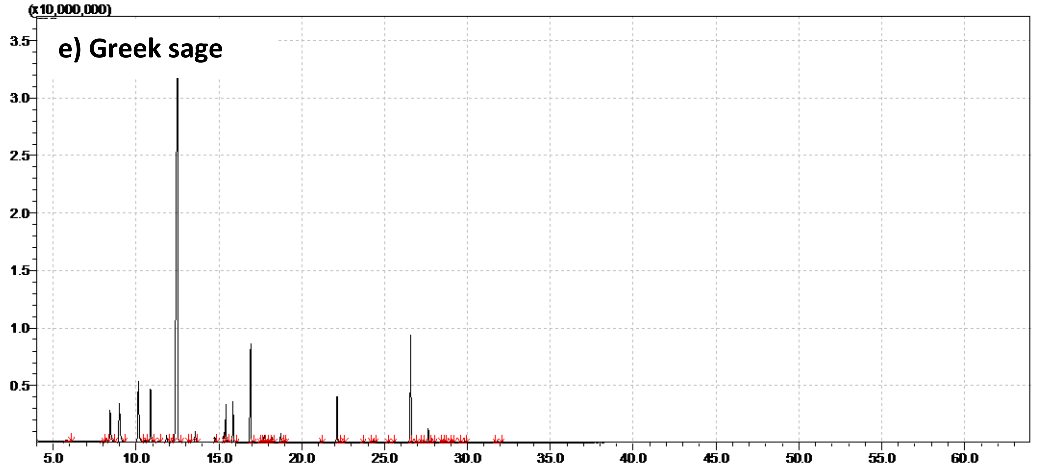
(x10,000,000)
e) Greek sage
3.5
3.0
2.5
2.0
1.5
1.0
0.5
5.0
10.0
15.0
20.0
25.0
30.0
35.0
40.0
45.0
50.0
55.0
60.0

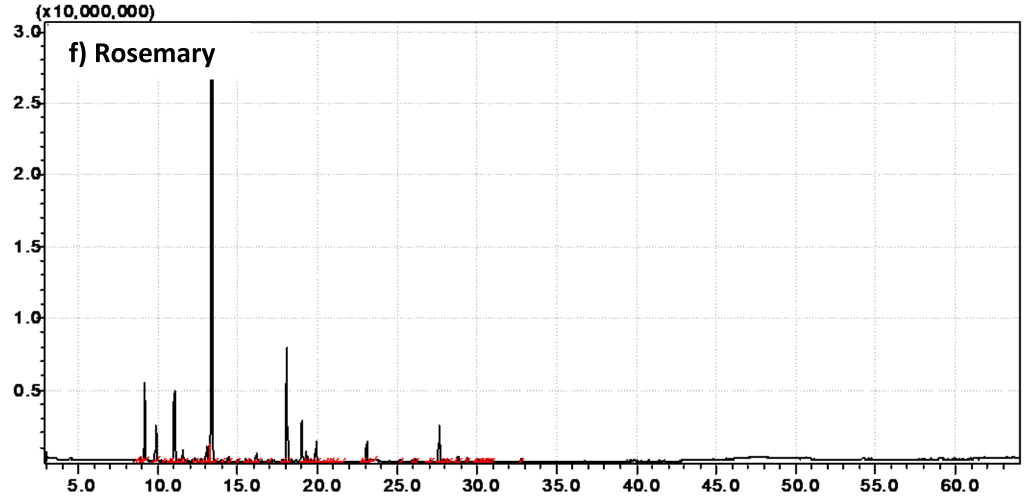
(x10,000,000)
f) Rosemary
3.0
2.5
2.0
1.5
1.0
0.5
5.0
10.0
15.0
20.0
25.0
30.0
35.0
40.0
45.0
50.0
55.0
60.0

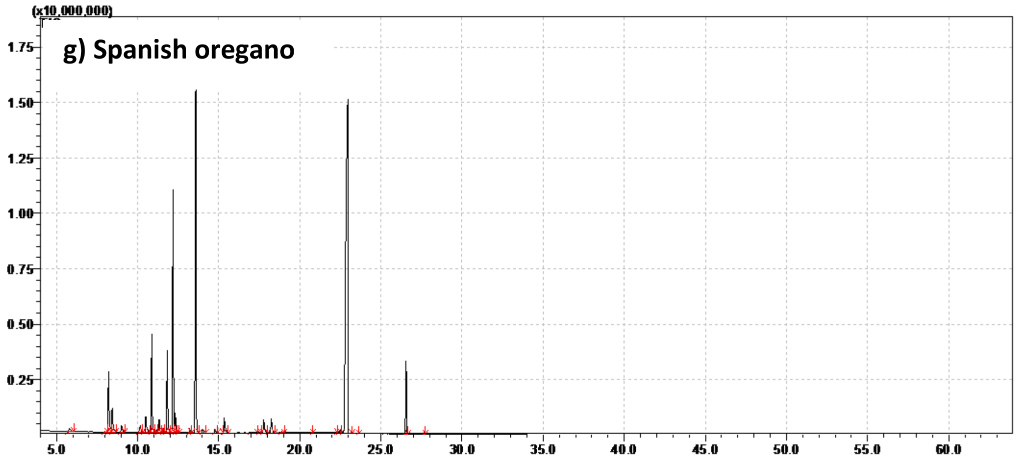
(x10,000,000)
g) Spanish oregano
1.75
1.50
1.25
1.00
0.75
0.50
0.25
5.0
10.0
15.0
20.0
25.0
30.0
35.0
40.0
45.0
50.0
55.0
60.0

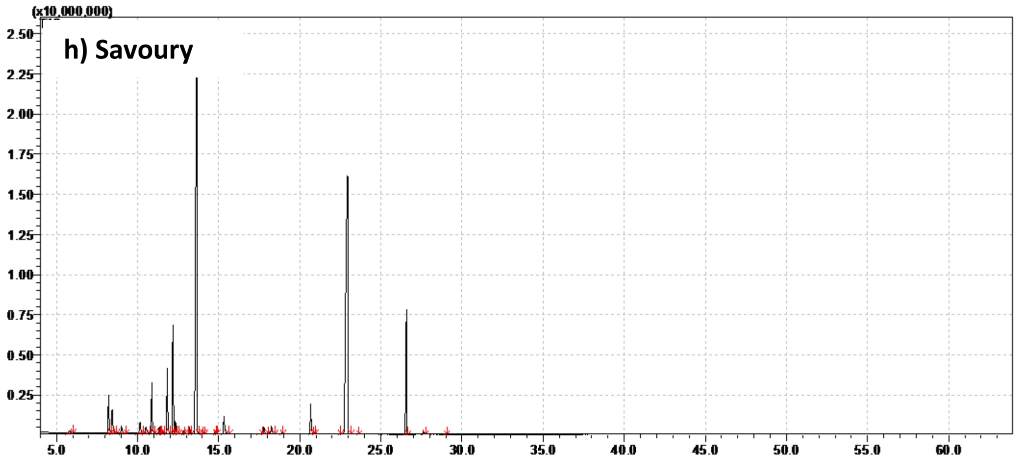
(x10,000,000)
h) Savoury
2.50
2.25
2.00
1.75
1.50
1.25
1.00
0.75
0.50
0.25
5.0
10.0
15.0
20.0
25.0
30.0
35.0
40.0
45.0
50.0
55.0
60.0

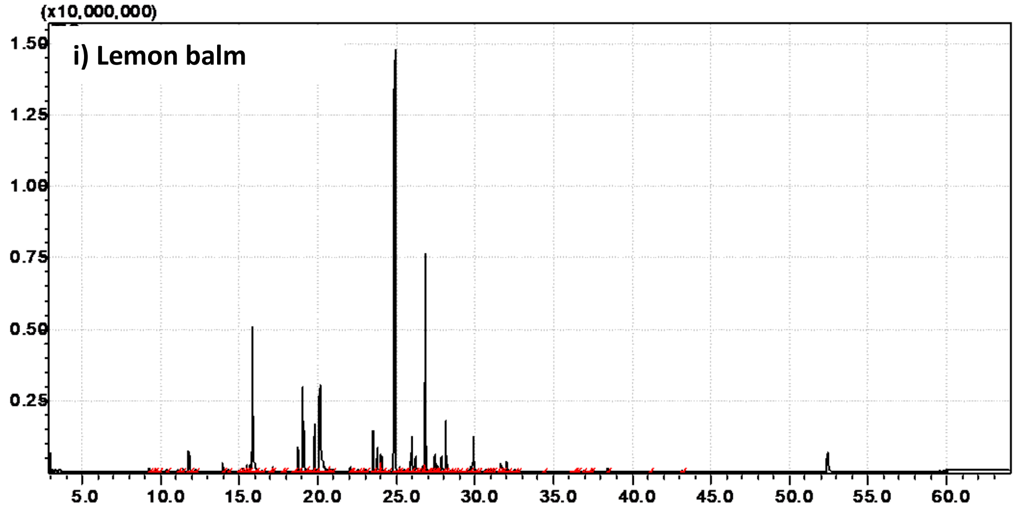
(x10,000,000)
i) Lemon balm
1.50
1.25
1.00
0.75
0.50
0.25
5.0
10.0
15.0
20.0
25.0
30.0
35.0
40.0
45.0
50.0
55.0
60.0

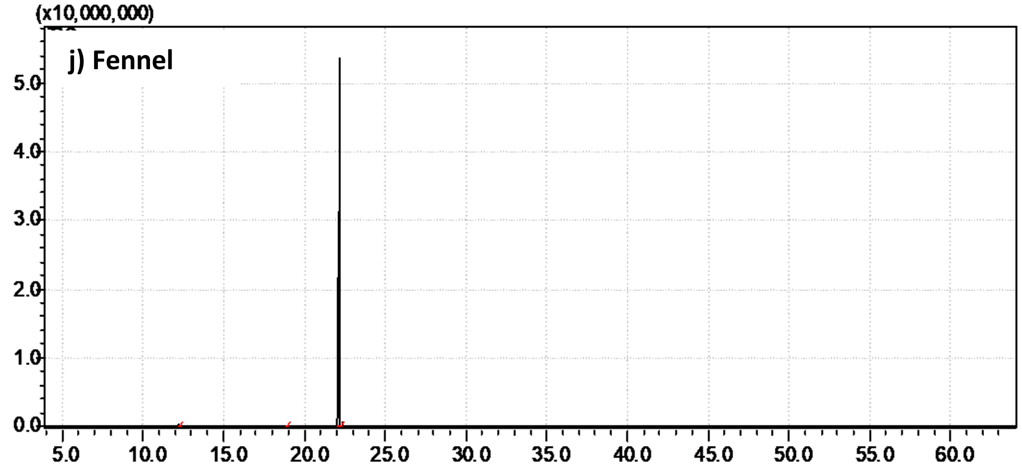
(x10,000,000)
j) Fennel
5.0
4.0
3.0
2.0
1.0
0.0
5.0 10.0 15.0 20.0 25.0 30.0 35.0 40.0 45.0 50.0 55.0 60.0

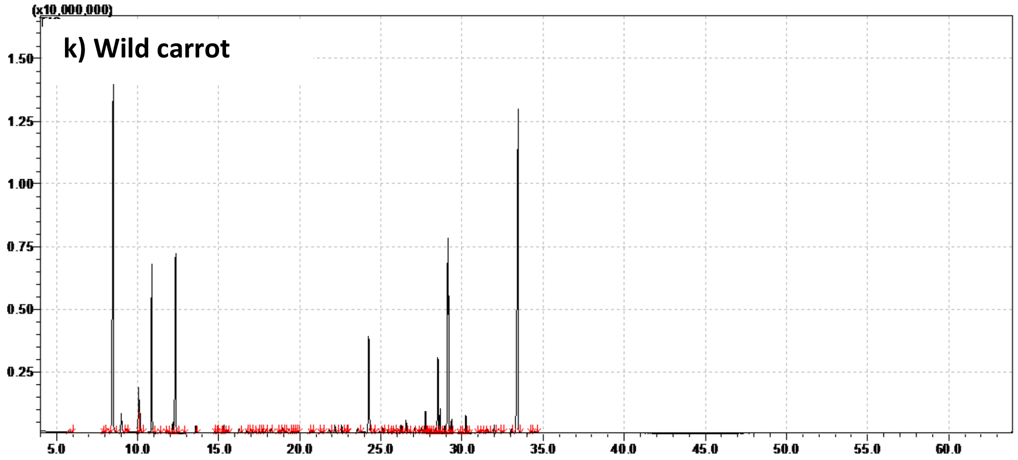
(x10,000,000)
k) Wild carrot
1.50
1.25
1.00
0.75
0.50
0.25
5.0 10.0 15.0 20.0 25.0 30.0 35.0 40.0 45.0 50.0 55.0 60.0

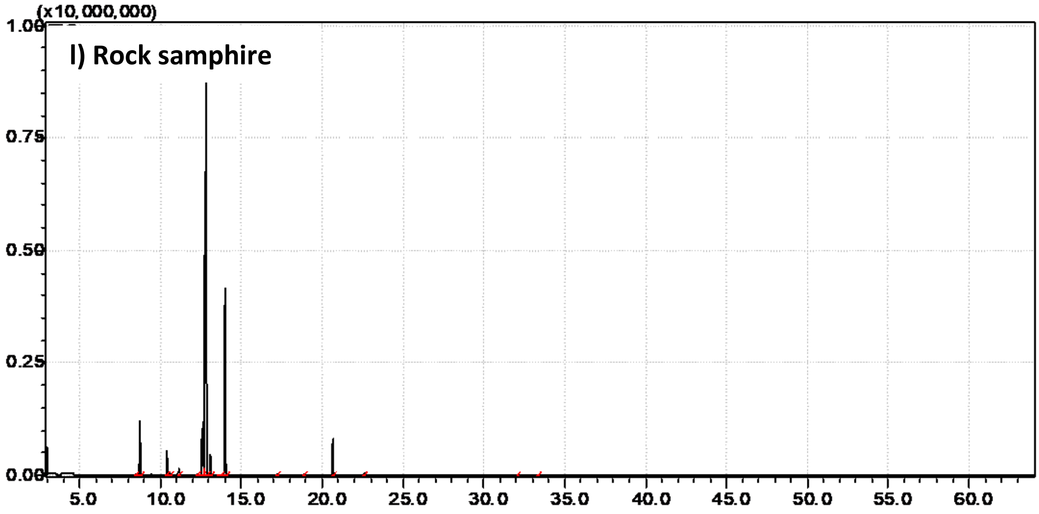
(x10,000,000)
l) Rock samphire
1.00
0.75
0.50
0.25
0.00
5.0 10.0 15.0 20.0 25.0 30.0 35.0 40.0 45.0 50.0 55.0 60.0

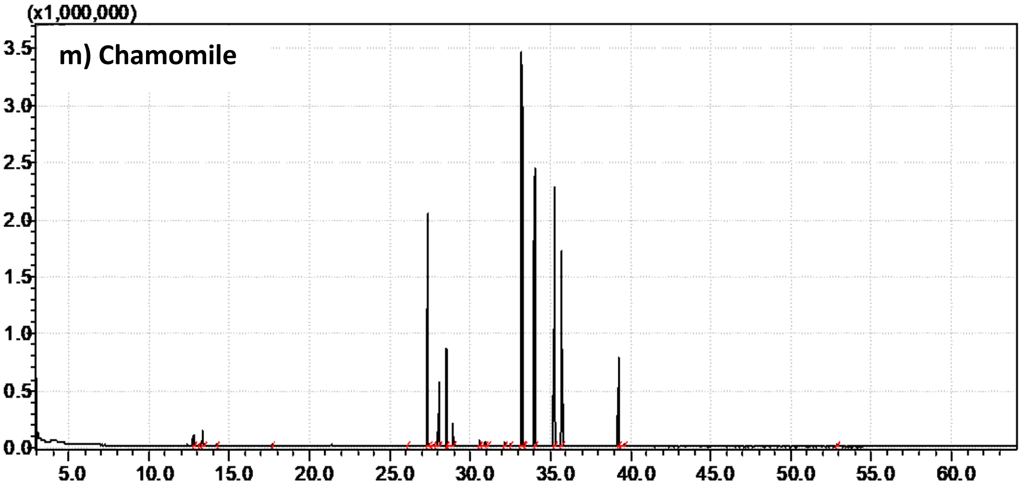

Figure S1. GC-MS chromatograms of a) pennyroyal from Ikaria, b) pennyroyal from Thessaloniki, c) lavender, d) Greek oregano, e) Greek sage, f) rosemary, g) Spanish oregano, h) savoury, i) lemon balm, j) fennel, k) wild carrot, l) rock samphire, and m) chamomile essential oils analyzed in the present study. Red colored arrows represent the identified peaks.

References

1. Shahmohamadi, R.; Sariri, R.; Rasa, M.; Ghafoori, H.; Aghamali, M.; Nasuti, S.; Tahery, M. Chemical composition and antimicrobial activity of flowering aerial parts *Mentha pulegium* from Gilan. *Pharmacologyonline* **2011**, *3*, 651–659.

2. Bouyahya, A.; Et-Touys, A.; Bakri, Y.; Talbaui, A.; Fellah, H.; Abrini, J.; Dakka, N. Chemical composition of *Mentha pulegium* and *Rosmarinus officinalis* essential oils and their antileishmanial, antibacterial and antioxidant activities. *Microb. Pathog.* **2017**, *111*, 41–49.

3. Zanjani, M.A.K.; Mohammadi, N.; Zojaji, M.; Bakhoda, H. Chemical composition of the essential oil of *Mentha pulegium* L. and its antimicrobial activity on *Proteus mirabilis*, *Bacillus subtilis* and *Zygosaccharomyces rouxii*. *J. Food Biosci. Technol.* **2015**, *5*, 31–40.

4. Milina, R.; Mustafa, Z.; Stanev, S.; Zvezdova, D.; Stoeva, S. Headspace gas chromatographic analysis of Bulgarian *Lavandula Angustifolia* Mill herbs . I. Optimization of the analysis conditions. *НАУЧНИ ТРУДОВЕ НА РУСЕНСКИЯ УНИВЕРСИТЕТ* **2012**, *51*, (9.1).

5. Zagorcheva, T.; Stanev, S.; Rusanov, K.; Atanassov, I. Comparitive GC/MS analysis of lavender (*Lavandula angustifolia* Mill.) inflorescence and essential oil volatiles. *J. Agric. Sci. Technol.* **2013**, *5*, 459–462.

6. Stanev, S.; Zagorcheva, T.; Atanassov, I. Lavender cultivation in Bulgaria – 21st century developments, breeding challenges and opportunities. *Bulg. J. Agric. Sci.* **2016**, *22*, 584–590.

7. Vokou, D.; Kokkini, S.; Bessiere, J.-M. Geographic variation of Greek oregano (*Origanum vulgare* ssp. *hirtum*) essential oils. *Biochem. Syst. Ecol.* **1993**, *21*, 287–295.

8. Adam, K.; Sivropoulou, A.; Kokkini, S.; Lanaras, T.; Arsenakis, M. Antifungal activities of *Origanum vulgare* subsp. *hirtum*, *Mentha spicata*, *Lavandula angustifolia*, and *Salvia fruticosa* essential oils against human pathogenic fungi. *J. Agric. Food Chem.* **1998**, *46*, 1739–1745.

9. Kokkini, S.; Karousou, R.; Hanlidou, E.; Lanaras, T. Essential oil composition of Greek (*Origanum vulgare* ssp. *hirtum*) and Turkish (*O. onites*) Oregano: A tool for their distinction. *J. Essent. Oil Res.* **2004**, *16*, 334–338.

10. Papageorgiou, V.; Gardeli, C.; Mallouchos, A.; Papaioannou, M.; Komaitis, M. Variation of the chemical profile and antioxidant behavior of *Rosmarinus officinalis* L. and *Salvia fruticosa* Miller grown in Greece. *J. Agric. Food Chem.* **2008**, *56*, 7254–7264.

11. Daferera, D.J.; Ziogas, B.N.; Polissiou, M.G. GC-MS analysis of essential oils from some Greek aromatic plants and their fungitoxicity on *Penicillium digitatum*. *J. Agric. Food Chem.* **2000**, *48*, 2576–2581.

12. Elmi, A.; Ventrella, D.; Barone, F.; Filippini, G.; Benvenuti, S.; Pisi, A.; Scozzoli, M.; Bacci, M.L. *Thymbra capitata* (L.) Cav. and *Rosmarinus officinalis* (L.) essential oils: *In vitro* effects and toxicity on swine spermatozoa. *Molecules* **2017**, *22*, 2162.

13. Pintore, G.; Usai, M.; Bradesi, P.; Juliano, C.; Boatto, G.; Tomi, F.; Chessa, M.; Cerri, R.; Casanova, J. Chemical composition and antimicrobial activity of *Rosmarinus officinalis* L. oils from Sardinia and Corsica. *Flavour Fragr. J.* **2002**, *17*, 15–19.

14. Salas, J.B.; Téllez, T.R.; Alonso, M.J.P.; Pardo, F.M.V.; Capdevila, M. de los Á.C.; Rodríguez, C.G. Chemical composition and antioxidant activity of the essential oil of *Thymbra capitata* (L.) Cav. in Spain. *Acta Bot. Gall.* **2010**, *157*, 55–63.

15. Delgado-Adámez, J.; Garrido, M.; Bote, M.E.; Fuentes-Pérez, M.C.; Espino, J.; Martín-Vertedor, D. Chemical composition and bioactivity of essential oils from flower and fruit of *Thymbra capitata* and *Thymus* species. *J. Food Sci. Technol.* **2017**, *54*, 1857–1865.

16. El Beyrouthy, M.; Arnold-Apostolides, N.; Cazier, F.; Najm, S.; Abou Jaoudeh, C.; Labaki, M.; Dhifi, W.; Abou Kais, A. Chemical composition of the essential oil of aerial parts of *Satureja Thymbra* L. growing wild in Lebanon. *Acta Hortic.* **2013**, *997*, 59–66.

17. Piras, A.; Cocco, V.; Falconieri, D.; Porcedda, S.; Marongiu, B.; Maxia, A.; Frau, M.A.; Gonçalves, M.J.; Cavaleiro, C.; Salgueiro, L. Isolation of the volatile oil from *Satureja thymbra* by supercritical carbon dioxide extraction: Chemical composition and biological activity. *Nat. Prod. Commun.* **2011**, *6*, 1523—1526.

18. Karousou, R.; Koureas, D.N.; Kokkini, S. Essential oil composition is related to the natural habitats: *Coridothymus capitatus* and *Satureja thymbra* in NATURA 2000 sites of Crete. *Phytochemistry* **2005**, *66*, 2668–2673.

19. Abdellatif, F.; Boudjella, H.; Zitouni, A.; Hassani, A. Chemical composition and antimicrobial activity of the essential oil from leaves of Algerian *Melissa officinalis* L. *EXCLI J.* **2014**, *13*, 772–781.

20. Taherpour, A.; Maroofi, H.; Rafie, Z.; Larijani, K. Chemical composition analysis of the essential oil of *Melissa officinalis* L. from Kurdistan, Iran by HS/SPME method and calculation of the biophysicochemical coefficients of the components. *Nat. Prod. Res.* **2012**, *26*, 152–160.

21. Diao, W.-R.; Hu, Q.-P.; Zhang, H.; Xu, J.-G. Chemical composition, antibacterial activity and mechanism of action of essential oil from seeds of fennel (foeniculum vulgare Mill.). *Food Control* **2014**, *35*, 109–116.

22. Zoubiri, S.; Baaliouamer, A.; Seba, N.; Chamouni, N. Chemical composition and larvicidal activity of Algerian *Foeniculum vulgare* seed essential oil. *Arab. J. Chem.* **2014**, *7*, 480–485.

23. Telci, I.; Demirtas, I.; Sahin, A. Variation in plant properties and essential oil composition of sweet fennel (*Foeniculum vulgare* Mill.) fruits during stages of maturity. *Ind. Crops Prod.* **2009**, *30*, 126–130.

24. Soković, M.; Stojković, D.; Glamočlija, J.; Ćirić, A.; Ristić, M.; Grubišić, D. Susceptibility of pathogenic bacteria and fungi to essential oils of wild *Daucus carota*. *Pharm. Biol.* **2009**, *47*, 38–43.

25. Özcan, M.M.; Chalchat, J.C. Chemical composition of carrot seeds (*Daucus carota* L.) cultivated in Turkey: Characterization of the seed oil and essential oil. *Grasas y Aceites* **2007**, *58*, 359–365.

26. Gonny, M.; Bradesi, P.; Casanova, J. Identification of the components of the essential oil from wild Corsican *Daucus carota* L. using 13C-NMR spectroscopy. *Flavour Fragr. J.* **2004**, *19*, 424–433.

27. Generalić Mekinić, I.; Blažević, I.; Mudnić, I.; Burčul, F.; Grga, M.; Skroza, D.; Jerčić, I.; Ljubenkov, I.; Boban, M.; Miloš, M.; et al. Sea fennel (*Crithmum maritimum* L.): phytochemical profile, antioxidative, cholinesterase inhibitory and vasodilatory activity. *J. Food Sci. Technol.* **2016**, *53*, 3104–3112.

28. Houta, O.; Akrout, A.; Najja, H.; Neffati, M.; Amri, H. Chemical composition, antioxidant and antimicrobial activities of essential oil from *Crithmum maritimum* cultivated in Tunisia. *J. Essent. Oil-Bearing Plants* **2015**, *18*, 1459–1466.

29. Baser, K.H.C.; Özek, T.; Demirci, B.; Saritas, Y. Essential oil of *Crithmum maritimum* l. from Turkey. *J. Essent. Oil Res.* **2000**, *12*, 424–426.

30. Pirzad, A.; Alyari, H.; Shakiba, M.R.; Zehtab-Salmasi, S.; Mohammadi, A. Essential oil content and composition of German Chamomile (*Matricaria chamomilla* L.) at different irrigation regimes. *J. Agron.* **2006**, *5*, 451–455.

31. Stanojevic, L.P.; Marjanovic-Balaban, Z.R.; Kalaba, V.D.; Stanojevic, J.S.; Cvetkovic, D.J. Chemical composition, antioxidant and antimicrobial activity of chamomile flowers essential oil (*Matricaria chamomilla* L.). *J. Essent. Oil-Bearing Plants* **2016**, *19*, 2017–2028.

32. Ayoughi, F.; Barzegar, M.; Sahari, M.A.; Naghdibadi, H. Chemical compositions of essential oils of *Artemisia dracunculus* L. and endemic *Matricaria chamomilla* L. and an evaluation of their antioxidative effects. *J. Agric. Sci. Technol.* **2011**, *13*, 79–88.
